# Non-coding and intergenic genetic variants of human arylamine *N*-acetyltransferase 2 (NAT2) gene are associated with differential plasma lipid and cholesterol levels and cardiometabolic disorders

**DOI:** 10.3389/fphar.2023.1091976

**Published:** 2023-04-03

**Authors:** Kyung U. Hong, Kennedy M. Walls, David W. Hein

**Affiliations:** Department of Pharmacology & Toxicology, Brown Cancer Center, University of Louisville School of Medicine, Louisville, KY, United States

**Keywords:** triglyceride, cholesterol, fatty acids, GWAS, haplotype, arylamine *N*-acetyltransferase 2, dyslipidemia

## Abstract

Arylamine N-acetyltransferase 2 (NAT2) is a phase II metabolic enzyme, best known for metabolism of aromatic amines and hydrazines. Genetic variants occurring in the NAT2 coding region have been well-defined and are known to affect the enzyme activity or protein stability. Individuals can be categorized into rapid, intermediate, and slow acetylator phenotypes that significantly alter their ability to metabolize arylamines, including drugs (e.g., isoniazid) and carcinogens (e.g., 4-aminobiphenyl). However, functional studies on non-coding or intergenic variants of *NAT2* are lacking. Multiple, independent genome wide association studies (GWAS) have reported that non-coding or intergenic variants of *NAT2* are associated with elevated plasma lipid and cholesterol levels, as well as cardiometabolic disorders, suggesting a novel cellular role of NAT2 in lipid and cholesterol homeostasis. The current review highlights and summarizes GWAS reports that are relevant to this association. We also present a new finding that seven, non-coding, intergenic *NAT2* variants (i.e., rs4921913, rs4921914, rs4921915, rs146812806, rs35246381, rs35570672, and rs1495741), which have been associated with plasma lipid and cholesterol levels, are in linkage disequilibrium with one another, and thus form a novel haplotype. The dyslipidemia risk alleles of non-coding *NAT2* variants are associated with rapid NAT2 acetylator phenotype, suggesting that differential systemic NAT2 activity might be a risk factor for developing dyslipidemia. The current review also discusses the findings of recent reports that are supportive of the role of NAT2 in lipid or cholesterol synthesis and transport. In summary, we review data suggesting that human *NAT2* is a novel genetic factor that influences plasma lipid and cholesterol levels and alters the risk of cardiometabolic disorders. The proposed novel role of NAT2 merits further investigations.

## 1 Introduction

Human populations separate into rapid, intermediate and slow acetylator phenotypes based on their capacity to catalyze the *N*-acetylation of aromatic amine and hydrazine drugs *via N*-acetyltransferase 2 (NAT2) as reviewed previously (McDonagh et al., 2014). NAT2 phenotypes have been associated with both ineffective dosing and drug toxicities with drugs commonly used for important conditions such as isoniazid for the prevention and treatment of tuberculosis (Hein and Millner, 2021). Similarly, NAT2 phenotypes have been associated with differential cancer risk following exposures to aromatic amine carcinogens metabolized by NAT2 (García-Closas et al., 2005; Agúndez, 2008; Moore et al., 2011; Hein, 2018).

Numerous investigations have documented that NAT2 acetylator phenotype is associated with single nucleotide polymorphisms (SNPs) in the 870 bp NAT2 coding exon. None of these SNPs have been shown to affect *NAT2* mRNA expression (Salazar-González et al., 2020); rather the SNPs in the NAT2 coding exon cause changes in NAT2 protein structure that can affect stability and/or substrate affinity (Walraven et al., 2008; Hein, 2009; Zhou et al., 2013).

## 2 Non-coding NAT2 variants associated with elevated plasma lipid and cholesterol levels

While previous studies on *NAT2* genetic polymorphism have focused on variants in the coding exon (see Introduction), *NAT2* variants in the non-coding or intergenic region have not been characterized, despite of the fact that many of these non-coding variants have been linked to pathological conditions by independent genome wide association studies (GWAS) (Table 1; Supplementary Table S1). In fact, all but one (i.e., rs1208) *NAT2* variants linked to different traits by previous GWAS are either intronic or intergenic variants. Among *NAT2* variants identified by GWAS, a total of 15 non-coding variants (out of 30) (i.e., rs11780610, rs11782802, rs13277394, rs1495741, rs1495743, rs1495745, rs1961456, rs34987019, rs35246381, rs35583283, rs4646248, rs4921913, rs4921914, rs4921915, and rs6997340) are independently linked to differential plasma lipid or cholesterol levels (Table 1; Supplementary Table S1).

**TABLE 1:** *NAT2* genetic variants associated with differential plasma lipid, cholesterol, or fatty acid levels.

Variant	Type	Risk allele	*p*-value	RAF^a^	Increase/decrease	Reported trait	Reference	Location^b^
rs1115784	Non-coding (intronic)	G	8 × 10^−7^	0.40	NA	Myocardial infarction	Hartiala et al. (2021)	8:18397900
rs11780610	Non-coding	C	9 × 10^−10^	0.38	↑	Apolipoprotein A1 levels	Richardson et al. (2020)	8:18402366
		T	2 × 10^−35^	0.62	↓	Triglycerides	Klarin et al. (2018)	
		T	2 × 10^−14^		NA	Mean corpuscular hemoglobin	Chen et al. (2020)	
rs11780884	Non-coding	A	2 × 10^−8^	0.32	↑	Free cholesterol levels in small HDL	Richardson et al. (2022)	8:18388543
rs11782802	Non-coding (intronic)	T	2 × 10^−14^	0.05	↑	Triglyceride levels	Sinnott-Armstrong et al. (2021)	8:18399145
rs1208	Coding	A	3 × 10^−6^	0.68	↑	Insulin resistance	Knowles et al. (2015)	8:18400806
rs13277394	Non-coding	T	9 × 10^−10^	0.01	↑	Triglyceride levels	Sinnott-Armstrong et al. (2021)	8:18408709
		T	9 × 10^−11^	0.01	↑	Total cholesterol levels	Sinnott-Armstrong et al. (2021)	
rs146812806	Non-coding	Ins	9 × 10^−14^	NR	↓	Mean corpuscular hemoglobin concentration	Chen et al. (2020)	8:18414994
rs1495741	Non-coding	A	4 × 10^−8^	0.65	↓	Triglyceride levels	Bentley et al. (2019)	8:18415371
		A	1 × 10^−19^		↓	Medication use (HMG-CoA reductase inhibitors)	Sakaue et al. (2021)	
		G	4 × 10^−14^	0.35	↑	Triglycerides	Teslovich et al. (2010)	
		G	2 × 10^−9^		↑	Cholesterol, total	Teslovich et al. (2010)	
		G	6 × 10^−6^		↑	Low density lipoprotein cholesterol levels	Hoffmann et al. (2018)	
		G	3 × 10^−8^		↑	Cholesterol, total	Willer et al. (2013)	
		G	4 × 10^−18^		↑	Monounsaturated fatty acid levels	Richardson et al. (2022)	
		G	1 × 10^−17^		↑	Medication use (HMG-CoA reductase inhibitors)	Wu et al. (2019)	
		G	4 × 10^−10^		↑	Apolipoprotein B levels	Richardson et al. (2020)	
rs1495743	Non-coding	C	1 × 10^−19^	0.65	↓	Triglyceride levels in current drinkers	de Vries et al. (2019)	8:18415790
		C	7 × 10^−11^		↓	LDL cholesterol	Sakaue et al. (2021)	
		G	3 × 10^−6^	0.35	↑	Low density lipoprotein cholesterol levels	Hoffmann et al. (2018)	
		G	5 × 10^−16^		↑	Triglycerides	Hoffmann et al. (2018)	
rs1495745	Non-coding	T	9 × 10^−9^	0.38	↓	Cholesteryl esters to total lipids ratio in very small VLDL	Richardson et al. (2022)	8:18405213
rs1961456	Non-coding (intronic)	A	7 × 10^−17^	0.59	↓	Total cholesterol levels	Spracklen et al. (2017)	8:18398199
rs34987019	Non-coding	T	2 × 10^−8^	0.73	↓	Total cholesterol levels	Klarin et al. (2018)	8:18416933
rs35246381	Non-coding	C	2 × 10^−13^	0.35	↓	Cholesteryl esters to total lipids ratio in medium VLDL	Richardson et al. (2022)	8:18415025
		C	2 × 10^−12^		↓	Cholesterol to total lipids ratio in medium VLDL	Richardson et al. (2022)	
		C	1 × 10^−10^		↑	Triglycerides to total lipids ratio in medium VLDL	Richardson et al. (2022)	
		C	1 × 10^−13^		↓	Mean corpuscular hemoglobin concentration	Vuckovic et al. (2020)	
rs35570672	Non-coding	C	3 × 10^−10^	0.65	↓	Mean corpuscular hemoglobin	Vuckovic et al. (2020)	8:18415125
rs35583283	Non-coding	G	1 × 10^−16^	0.71	↓	Familial combined hyperlipidemia defined by Consensus criteria	Trinder et al. (2022)	8:18396999
		C	3 × 10^−9^	0.39	↑	Triglycerides to total lipids ratio in large LDL	Richardson et al. (2022)	
		T	2 × 10^−7^	0.61	↓	Coronary artery disease	van der Harst and Verweij (2018)	
rs4921913	Non-coding	C	5 × 10^−8^	0.35	↑	Metabolic syndrome	Lind, (2019)	8:18414867
		C	2 × 10^−10^		↑	Cholesterol levels in large VLDL	Richardson et al. (2022)	
		C	3 × 10^−11^		↑	Triglyceride levels in large VLDL	Richardson et al. (2022)	
rs4921914	Non-coding	T	7 × 10^−8^	0.65	↓	Triglyceride levels	Bentley et al. (2019)	8:18414928
		T	9 × 10^−12^		NA	Mean corpuscular hemoglobin concentration	Chen et al. (2020)	
		T	2 × 10^−20^		↓	Triglyceride levels	Spracklen et al. (2017)	
rs4921915	Non-coding	A	2 × 10^−10^	0.65	↓	Triglycerides	Sakaue et al. (2021)	8:18414956
		A	3 × 10^−22^		↓	Total cholesterol levels	Sakaue et al. (2021)	
		G	2 × 10^−16^	0.35	↑	Total triglycerides levels	Richardson et al. (2022)	
		G	2 × 10^−8^		↑	Polyunsaturated fatty acid levels	Richardson et al. (2022)	
rs6997340	Non-coding	T	5 × 10^−9^	0.43	↑	Coronary artery disease	van der Harst and Verweij (2018)	8:18429487
rs721399	Non-coding	T	9 × 10^−13^	0.56	↓	Mean corpuscular hemoglobin	Chen et al. (2020)	8:18401856
rs73207888	Non-coding	T	3 × 10^−8^	0.14	↑	Medication use (calcium channel blockers)	Wu et al. (2019)	8:18425421

RAF, relative allele frequency; NR, not reported; NA, not available; Ins, insertion.

^a^ Based on 1,000 Genomes Project (phase 3).

^b^ Human GRCh38/hg38.

One intergenic variant, rs1495741 (chr8: 18415371; GRCh38/hg38), located approximately 14 kb downstream of the NAT2 coding region, has been linked to more than 65 traits by multiple, independent GWAS (Table 1; Supplementary Table S1). Most of these GWAS-linked traits are related to plasma lipid, fatty acid, and cholesterol levels (i.e., 58 out of 68 traits). For instance, the “G” allele of rs1495741 (rs1495741-G) (relative allele frequency [RAF] 0.35) has been linked to elevated levels of multiple plasma lipid profiles, including triglyceride, phospholipids, and fatty acids (Table 1; Supplementary Table S1). Consistent with this finding, its other major allele, rs1495741-A (RAF 0.65), has been linked to a *decreased* level of plasma triglyceride (Table 1; Supplementary Table S1). In addition, rs1495741-G is independently associated with elevated plasma cholesterol (free or total) levels and also with elevated ratio of cholesteryl esters-to-total lipids in small LDL, as well as apolipoprotein B level (Table 1; Supplementary Table S1). With respect to the ratio of cholesterol-to-total lipids or cholesteryl esters-to-total lipids in HDL, rs1495741-G is associated with a significantly decreased ratio. Moreover, not only is rs1495741-G linked to an elevated level of total free fatty acids in plasma, but also to increased monosaturated or saturated free fatty acid levels (Table 1; Supplementary Table S1). Another intergenic variant, rs4921913 (chr8:18414867), which is located approximately 500 bp from rs1495741, has been linked to 30 traits so far. The common traits (i.e., 26 out of 30 reported traits) linked to the “C” allele of rs4921913 include significant increases in plasma lipid, fatty acid, and cholesterol levels (Table 1; Supplementary Table S1). Similarly, thirteen other non-coding variants of *NAT2* (i.e., rs11780610, rs11782802, rs13277394, rs1495743, rs1495745, rs1961456, rs34987019, rs35246381, rs35583283, rs4646248, rs4921914, rs4921915, and rs6997340) are linked to differential plasma lipid and/or cholesterol levels. Taken together, half of non-coding or intergenic variants of *NAT2* associate with dyslipidemia.

## 3 Non-coding *NAT2* variants are associated with increased risks of cardiometabolic disorders

It is often difficult to assess if elevated lipid or cholesterol levels conferred by *NAT2* genetic variants can ultimately lead to clinically meaningful outcomes. However, several GWAS findings support the idea that they contribute to clinical consequences. For instance, rs4646248-T (RAF 0.61) which is associated with elevated triglycerides to total lipids ratio, is also linked to coronary artery disease by two independent GWAS (Table 1; Supplementary Table S1), whereas its other major allele, r4646248-C (RAF 0.39), is linked to *decreased* coronary artery disease risk by two independent GWAS (Table 1; Supplementary Table S1). Similarly, rs1115784-G (RAF 0.40) is linked to increased risk of myocardial infarction (Table 1; Supplementary Table S1). A couple of coding (rs1208-A; RAF 0.68) and non-coding (rs4921913-C; RAF 0.35) *NAT2* SNPs also have been linked to metabolic disorders, i.e., insulin resistance and metabolic syndrome, respectively (Table 1; Supplementary Table S1). Although rs1208, a coding SNP, has not been linked to differential plasma lipid or cholesterol levels by previous GWAS, rs4921913-C is linked to elevated plasma cholesterol, triglyceride, total lipid, and phospholipid levels by numerous GWAS (Table 1; Supplementary Table S1).

Additionally, a recent GWAS by Trinder et al. (2022) reported that “G” allele of an intronic SNP, rs35583283 (RAF 0.71), is associated with familial combined hyperlipidemia (Table 1; Supplementary Table S1). A GWAS study published in 2014 investigated shared molecular pathways and gene networks for cardiovascular disease and type 2 diabetes in women of three different ethnic backgrounds (Chan et al., 2014). Among Caucasian women, for the combined phenotype (cardiovascular disease + type 2 diabetes), a *NAT2* SNP, rs7825609, reached genome-wide significance in the standard GWAS analysis (Table 1; Supplementary Table S1) (Chan et al., 2014). Unlike other non-coding variants discussed above, rs7825609 is located approximately 2.5 kb upstream of the *NAT2* coding region and approximately 0.4 kb downstream of a long non-coding RNA gene, *ENSG00000285624* (chr8:18386311–18388323).

Non-coding *NAT2* variants also have been associated with increased cardiovascular medication usage, which indirectly supports that dyslipidemia conferred by risk alleles of *NAT2* variants can result in clinically pathological conditions. For example, rs73207888-T (RAF 0.14) is associated with increased usage of calcium channel blockers (Table 1; Supplementary Table S1) which are commonly prescribed for cardiovascular conditions, such as hypertension (Eisenberg et al., 2004). In addition, rs1495741-G, which is linked to elevated cholesterol levels by multiple, independent GWAS, is also associated with increased incidence of HMG-CoA reductase inhibitor use (Table 1; Supplementary Table S1), a popular class of drugs used to treat hypercholesterolemia and prevent development of atherosclerosis (Bansal and Cassagnol, 2022).

In summary, some of the *NAT2* variants, which are associated with elevated plasma lipid and cholesterol levels, also have been identified as risk alleles for cardiometabolic disorders, including coronary artery disease, myocardial infarction, and metabolic syndrome, as well as increased cardiovascular drug use. Collectively, these data suggest that elevated plasma lipid or cholesterol levels conferred by the *NAT2* risk alleles can culminate in development of cardiometabolic disorders. These GWAS findings are consistent with the well-established fact that hyperlipidemia or hypercholesterolemia is a major risk factor for cardiometabolic disease (Nelson, 2013).

## 4 Identification of a new *NAT2* haplotype/locus linked to plasma lipid and cholesterol levels

**FIGURE 1:**
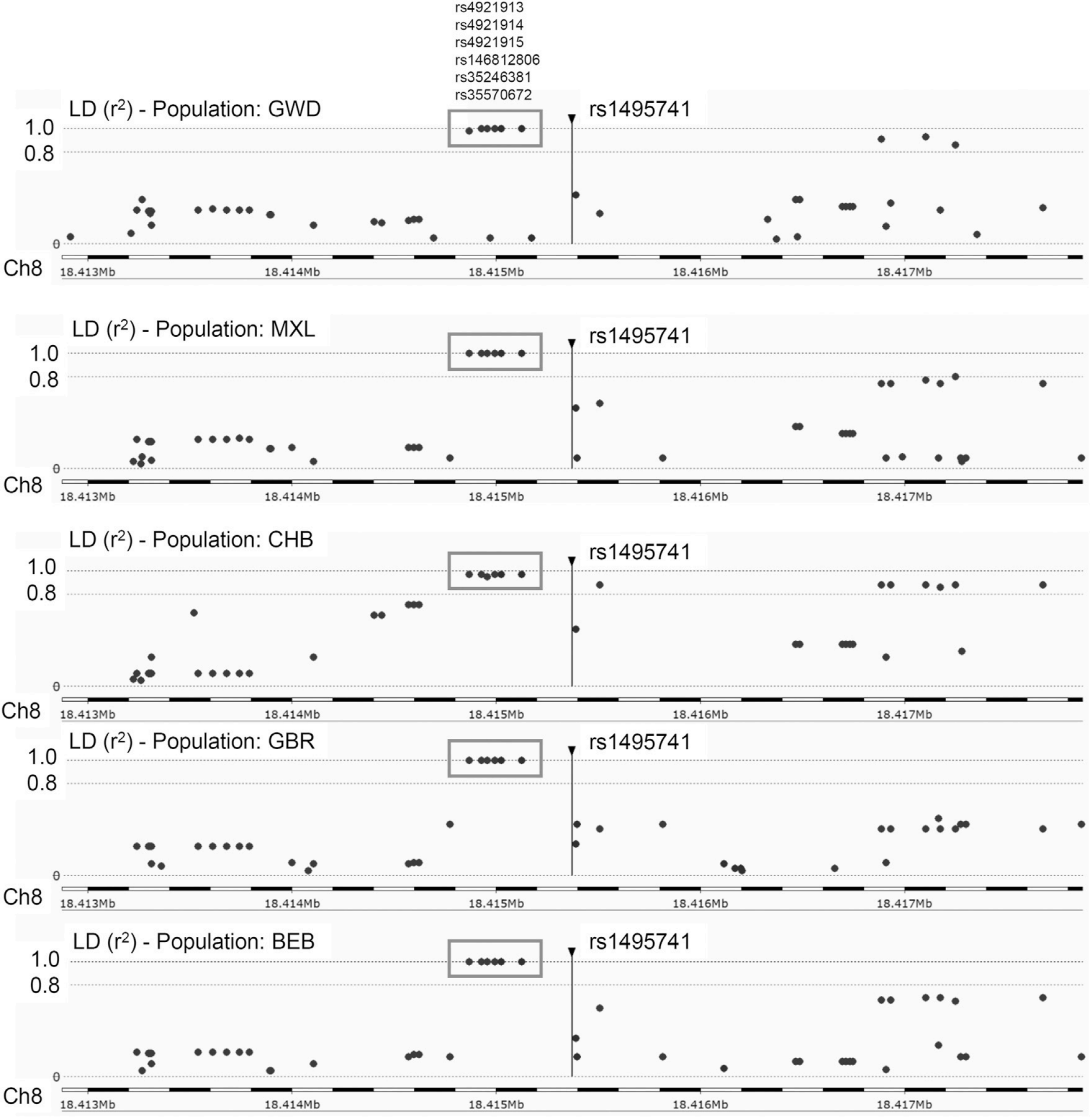
Linkage disequilibrium (LD) plots for rs1495741 in representative populations from 1000 Genomes Project. The plots show variants that are (or are not) in linkage disequilibrium with rs1495741. Each dot represents a variant (in a surrounding 5-kb region). The longitudinal dotted lines show LD *r* ^2^ value of 1.0 and 0.8, with respect to rs1495741. Numbers on the bottom axis show the location on human chromosome 8 (GRCh38/hg38). The position of rs1495741 is represented by a vertical line. Six variants in the grey box (i.e., rs4921913, rs4921914, rs4921915, rs146812806. rs35246381, and rs35570672) are in linkage disequilibrium with rs1495741 with *r* ^2^ value of ∼1.0 in multiple human populations from 1000 Genomes Project (1000 Genomes Project Consortium et al., 2015). GWD, Gambian in Western Division; MXL, Mexican Ancestry in Los Angeles; CHB, Han Chinese in Beijing, China; GBR, British in England and Scotland; BEB, Bengali in Bangladesh. The plots were generated using Ensembl’s linkage disequilibrium calculator (Linkage Disequilibrium Calculator - Homo_sapiens - Ensembl genome browser 106, 2022).

Previously, it was reported that two variants associated with hyperlipidemia, rs1495741 and rs4921914, are in linkage disequilibrium (LD) (Fathzadeh et al., 2018). We examined if other *NAT2* genetic variants linked to hyperlipidemia (Table 1; Supplementary Table S1) are in LD with one another, using tools available at Ensembl genome browser and data from 1000 Genomes Project (Fairley et al., 2020; Ensembl Genome Browser, 2022; Linkage Disequilibrium Calculator - Homo_sapiens - Ensembl genome browser 106, 2022), Surprisingly, seven variants (i.e., rs1495741, rs4921913, rs4921914, rs4921915, rs146812806, rs35246381, and rs35570672) showed LD *r* ^2^ value of approximately 1.0 with one another in all human populations from 1000 Genomes Project (Figure 1 and data not shown). Six of them are SNPs, whereas rs146812806 is an insertion–deletion mutation (i.e., “indel”). Interestingly, these seven variants are all non-coding, intergenic variants located approximately 14 kb downstream of NAT2 coding region and within ∼0.5 kb from one another (ch8: 18272377–18272881) (Figure 1; Table 3). This implies that these seven closely located variants represent a haplotype (a.k.a, linked polymorphism). To our knowledge, such finding has never been reported previously. Another SNP, rs34987019 (ch8:18,416,933), associated with plasma cholesterol level (Table 1; Supplementary Table S1), also is in LD with rs14957141 in certain populations (LD *r* ^2^ = 0.36–0.94) (Linkage Disequilibrium Calculator - Homo_sapiens - Ensembl genome browser 106, 2022). However, according to our *in silico* analysis, the insulin resistance risk allele, rs1208, or a myocardial infarction risk allele, rs1115784, (Table 1; Supplementary Table S1), were not in LD (*r* ^2^ < 0.8) with any of the seven variants in the haplotype (data not shown).

Five out of the seven variants (rs1495741, rs35246381, rs4921915, rs4921914, and rs4921913) in this haplotype have been reported to be associated with elevated plasma and cholesterol levels by previous GWAS (Table 1; Supplementary Table S1). Although rs35570672 and rs146812806 have never been associated with plasma lipid and cholesterol levels, they are associated with differential urinary and serum metabolite levels (Table 2; Supplementary Table S2) that are indicative of systemic NAT2 activity (see discussions below in Section 6). It is notable that both rs35570672 and rs146812806 have been associated with differential levels of mean corpuscular hemoglobin, a parameter measured in complete blood count (CBC). Components of the CBC, such as the red blood cell, platelet counts, hemoglobin and hematocrit values, are associated with coronary artery disease and can be used in combination with the white blood cell (WBC) count to predict the risk (Madjid and Fatemi, 2013). One study by Anderson et al. (2007) showed that CBC-derived risk scores for coronary artery disease can be further improved by incorporating values on hematocrit, mean corpuscular volume, red blood cell distribution width, mean corpuscular hemoglobin concentration, and platelet and WBC counts. We speculate that the association between two *NAT2* variants (rs35570672 and rs146812806) and mean corpuscular hemoglobin level is reflective of the dyslipidemia and coronary artery disease risk associated with the haplotype. This is supported by the fact that rs35570672-T and rs146812806-TGCCTG (insertion) alleles are co-segregated with the rest of the variants in the seven-variant haplotype, including rs1495741-G and s4921913-C (Figure 1; Table 3). As previously mentioned, rs1495741-G is not only linked to elevated plasma cholesterol, triglyceride, total lipid, and phospholipid levels, but also to metabolic syndrome (Table 1; Supplementary Table S1), which implies that the seven-variant haplotype is a risk locus for dyslipidemia as well as cardiometabolic disorders.

**TABLE 2:** *NAT2* genetic variants associated with differential urinary or serum metabolite levels and other traits.

Variant	Type	Risk Allele	*p*-value	RAF^a^	Increase/decrease	Reported trait	Reference	Location^b^
rs11784251	Non-coding	G	2 × 10^−17^	0.56	↑	Blood metabolite levels (*N*-acetylputrescine)	Rhee et al. (2022)	8:18402503
rs146812806	Non-coding	Ins	9 × 10^−32^	0.35	↑	Serum metabolite levels (5-acetylamino-6-amino-3-methyluracil)	Feofanova et al. (2020)	8:18414994
rs1495741	Non-coding	NR	4 × 10^−11^	–	NA	Bladder cancer	Rothman et al. (2010)	8:18415371
		A	1 × 10^−27^	0.65	↑	Blood metabolite levels (1-methylurate)	Shin et al. (2014)	
		A	2 × 10^−10^		NA	Bladder cancer	Figueroa et al. (2014)	
		A	7 × 10^−11^		NA	Liver injury in anti-tuberculosis drug treatment	Suvichapanich et al. (2019)	
		A	1 × 10^−24^		↓	*N*-acetylputrescine levels (blood)	Rhee et al. (2022)	
		A	2 × 10^−15^		↓	4-acetamidobutanoate levels (blood)	Rhee et al. (2022)	
rs1495743	Non-coding	NR	6 × 10^−16^	-	↓	Serum metabolite levels (1-methylxanthine)	Krumsiek et al. (2012)	8:18415371
		NR	7 × 10^−9^	-	↑	4-acetamidobutanoate levels (serum)	Yet et al. (2016)	
		G	2 × 10^−40^	0.35	↓	Metabolic traits (SM-7 + 11 other traits)	Suhre et al. (2011a)	
rs35246381	Non-coding	C	3 × 10^−35^	0.35	↑	Urinary metabolite levels in chronic kidney disease (*N*-acetylputrescine)	Schlosser et al. (2020)	8:18415025
		C	2 × 10^−25^		↑	Urinary metabolite modules in chronic kidney disease (4-acetamidobutanoate, allo-threonine, *N*-acetylputrescine)	Schlosser et al. (2020)	
		C	1 × 10^−72^		↑	Urinary metabolite levels in chronic kidney disease (5-acetylamino-6-formylamino-3-methyluracil)	Schlosser et al. (2020)	
		C	7 × 10^−128^		↑	Serum metabolite levels (5-acetylamino-6-formylamino-3-methyluracil)	Feofanova et al. (2020)	
		C	6 × 10^−24^		↑	Urinary metabolite levels in chronic kidney disease (5-acetylamino-6-amino-3-methyluracil)	Schlosser et al. (2020)	
rs35570672	Non-coding	T	4 × 10^−40^	0.35	↓	Serum metabolite levels (1-methylxanthine)	Feofanova et al. (2020)	8:18415125
		T	1 × 10^−100^		↑	Serum metabolite levels (*N*-acetylputrescine)	Feofanova et al. (2020)	
rs4921913	Non-coding	NR	7 × 10^−9^	-	↓	1-methylxanthine levels (serum)	Yet et al. (2016)	8:18414867
		NR	2 × 10^−19^	-	↑	5-acetylamino-6-formylamino-3-methyluracil levels (serum)	Bar et al. (2020)	
		C	6 × 10^−44^	0.35	↑	Serum metabolite levels (4-acetamidobutanoate)	Feofanova et al. (2020)	
rs1495743	Non-coding	T	3 × 10^−47^	0.35	↓	Blood metabolite ratios (4-acetamidobutanoate/N1-methyladenosine)	Shin et al. (2014)	8:18414867
rs4921914	Non-coding	NR	6 × 10^−18^	-	↑	*N*-acetylputrescine levels (serum)	Bar et al. (2020)	8:18414928
		C	1 × 10^−28^	0.35	NA	Urinary metabolites (Formate/succinate ratio)	Suhre et al. (2011b)	
		C	1 × 10^−11^		↓	Urinary metabolite levels in chronic kidney disease (1-methylurate)	Schlosser et al. (2020)	
		T	1 × 10^−60^	0.65	↑	Blood metabolite levels (1-methylxanthine)	Shin et al. (2014)	
rs4921915	Non-coding	G	1 × 10^−19^	0.35	↑	Urinary metabolite levels in chronic kidney disease (4-acetamidobutanoate)	Schlosser et al. (2020)	8:18414956
rs7006687	Non-coding	T	2 × 10^−6^	0.57	NA	QT interval (drug interaction; sulfonylurea hypoglycemic agents)	Avery et al. (2014)	8:18376073
rs721399	Non-coding	T	4 × 10^−58^	0.56	↓	Blood metabolite levels (4-acetamidobutanoate)	Shin et al. (2014)	8:18401856
		T	2 × 10^−10^		↓	4-acetamidobutanoate levels (blood)	Rhee et al. (2022)	

RAF, relative allele frequency; NR, not reported; NA, not available; Ins, insertion.

^a^ Based on 1,000 Genomes Project (phase 3).

^b^ Human GRCh38/hg38.

**TABLE 3:** Seven-variant dyslipidemia haplotype in *NAT2.*

Variant	Location (hg38)	Distance (bp)	Genotype	
**rs1495741**	8:18272881	0	**G**	**A**
rs35570672	8:18272635	246	T	C
rs35246381	8:18272535	346	C	T/A
rs146812806	8:18272503-18272504	378	TGCCTG	–
rs4921915	8:18272466	415	G	A
rs4921914	8:18272438	443	C	T/G
rs4921913	8:18272377	504	C	T
		RAF	0.35	0.65
		Dyslipidemia risk allele	√	
		Acetylator phenotype^a^	Rapid	Slow

RAF, relative allele frequency.

**FIGURE 2:**
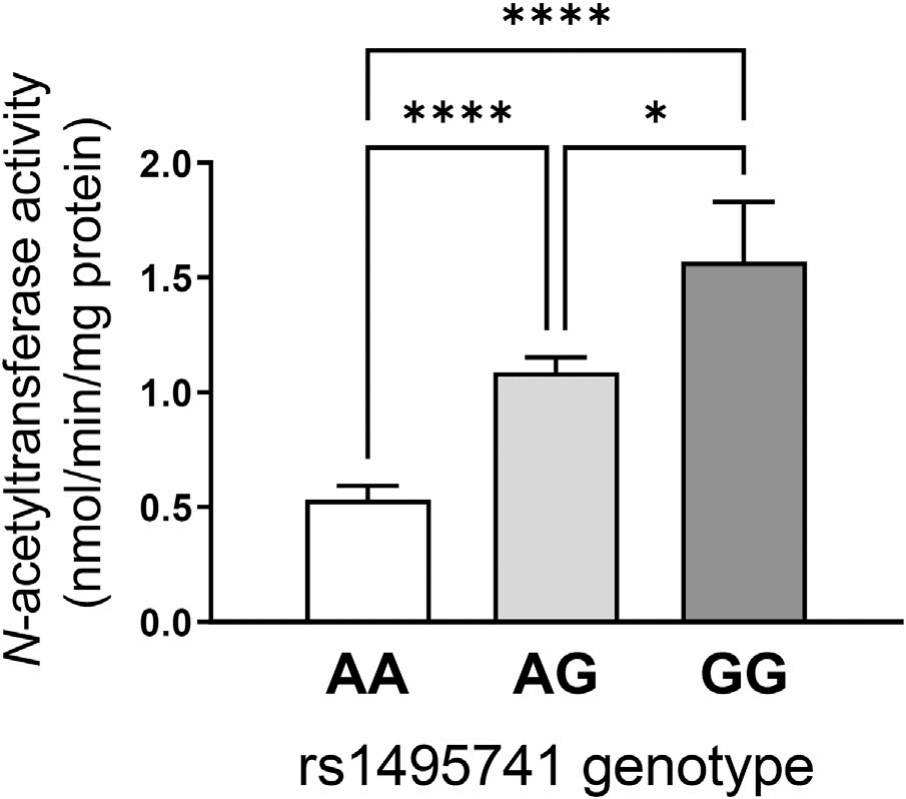
rs1495741 genotype-dependent changes in NAT2 activity in cryopreserved human hepatocytes. Bar graphs showing NAT2 activities (measured by *N*-acetylation of sulfamethazine) in cryopreserved human hepatocytes from 154 individuals of European background. Data was categorized by genotypes of rs1495741, a non-coding *NAT2* SNP (i.e., AA, AG, and GG). The “G” allele represents the dyslipidemia allele (see Table 1 and Supplementary Table S1). One-way ANOVA analysis was done followed by Tukey’s multiple comparison test. *, *p* < 0.05. ****, *p* < 0.0001. The figure was modified from data presented in our previous study (García-Closas et al., 2011).

^a^ Based on data available for rs1495741 (see Figure 2).

These findings have the following, important implications: 1) the risk of hyperlipidemia and metabolic syndrome is conferred by this newly discovered haplotype; 2) approximately 5%–40% of the world population (i.e., homozygotes for rs1495741-G) may be at a greater risk of developing dyslipidemia and cardiometabolic disease.

## 5 Linkage disequilibrium between other variants

*NAT2* SNPs, rs11780610 and rs4646248, which are linked with differential triglyceride levels and coronary artery disease risk, respectively (Table 1; Supplementary Table S1), are in linkage disequilibrium (LD *r* ^2^ > 0.9) based on data from 1000 Genomes Project (Linkage Disequilibrium Calculator - Homo_sapiens - Ensembl genome browser 106, 2022). These two SNPs exhibit variable linkage disequilibrium with rs1495741 (of the seven-variant dyslipidemia haplotype; Table 3), depending on the population, with *r* ^2^ value ranging between 0.08 and 0.84.

SNPs, rs11784251 and rs1390360, which are linked with differential serum *N*-acetylputrescine level and serum albumin level, respectively (Table 2; Supplementary Table S2), are in linkage disequilibrium (LD *r* ^2^ > 0.9). These two SNPs exhibit variable linkage disequilibrium with rs1495741, depending on the population, with *r* ^2^ value ranging between 0.28 and 0.85. In addition, rs4646248 and rs1495747 (Tables 1, 2) also are in linkage disequilibrium in most populations from the 1000 Genomes Project (*r* ^2^ = 0.2–1.0). Similarly, they exhibit variable linkage disequilibrium with rs1495741, depending on the population, with *r* ^2^ value ranging between 0.28 and 0.87.

## 6 Dyslipidemia risk alleles are linked to rapid acetylator phenotype

How do non-coding or intergenic *NAT2* variants confer risk of dyslipidemia and cardiometabolic disease? Although experimental evidence is lacking, the dyslipidemia risk alleles have been associated with *rapid* NAT2 acetylator phenotypes (i.e., relatively high NAT2 activity). Our lab previously measured NAT2 activity in 154 human hepatocytes from individuals of European background according to genotypes at rs1495741 (i.e., A/A, G/A, and G/G) and found that hepatocytes from individuals that carry the G allele had significantly higher NAT2 activity compared to those with the A allele (Figure 2). Accordingly, the G/G genotype at rs1495741 accurately predicted the rapid acetylator phenotype (García-Closas et al., 2011) (Figure 2). In other words, the dyslipidemia risk allele, rs1495741-G, is associated with relatively *high* NAT2 activity in human hepatocytes, although the mechanism is presently unknown.

Variants that are linked to differential urinary or serum metabolite levels (Table 2; Supplementary Table S2) also support the idea that the dyslipidemia risk alleles are associated with *high* NAT2 activity. Multiple, non-coding *NAT2* variants, including all seven variants in the haplotype (rs721399, **rs4921913**, **rs4921914**, **rs4921915**, **rs146812806**, **rs35246381**, **rs35570672**, **rs1495741**, and rs1495743; variants in the seven-variant haplotype in bold) associate with differential levels of urinary and serum metabolites, including 5-acetylamino-6-amino-3-methyluracil (AAMU), 5-acetylamino-6-formylamino-3-methyluracil (AAFU), 1-methylxanthine, 1-methylurate, 4-acetamidobutanoate, and *N*-acetylputrescine (Table 2; Supplementary Table S2). For instance, two risk alleles in the dyslipidemia risk haplotype, rs35246381-C and rs146812806-insertion, are both associated with a significant increase in AFMU and AAMU, respectively (Table 2; Supplementary Table S2). These metabolites are both major intermediates in caffeine metabolism involving NAT2 (Relling et al., 1992; Welfare et al., 2000). Accordingly, the elevated serum level of these caffeine metabolites associated with rs35246381-C and rs146812806-insertion may be attributed to relatively high NAT2 activity. Consistent with this finding, rs35246381-C, and rs146812806-insertion are in linkage disequilibrium with rs1495741-G, and thus are, both, associated with the *rapid* NAT2 acetylator phenotype (Figure 2; Table 3).

Additionally, rs35570672-T, which is associated with hyperlipidemia (Table 1; Supplementary Table S1), is also associated with elevated level of *N*-acetylputrescine in serum (Table 2; Supplementary Table S2). Putrescine is a recently identified endogenous substrate of human NAT2 (Conway et al., 2020; Salazar-Gonzalez and Hein, 2022), and *N*-acetylputrescine can form by acetylation of putrescene by human NAT2. Thus, the significant increase in serum *N*-acetylputrescine may reflect relatively high, systemic NAT2 activity. This, again, supports that rs35570672-T, a risk allele for hyperlipidemia, is linked to *rapid* NAT2 acetylator phenotype (Table 3).

In summary, some of the risk alleles for dyslipidemia or cardiometabolic disease also have been associated with differential urinary or serum metabolite levels which are indicative of relative NAT2 activity. Analysis of these genetic variants, that are associated with both dyslipidemia and differential serum or urine metabolite levels (e.g., rs35570672-T) (Tables 1, 2), demonstrate that the risk alleles of *NAT2* for dyslipidemia are associated with *higher* NAT2 activity (i.e., rapid NAT2 acetylator phenotype). Importantly, this implies that 1) plasma lipid or cholesterol levels may be, in part, determined by the level of enzymatic activity of NAT2 and 2) NAT2 may play a novel role in lipid and cholesterol homeostasis.

## 7 Evidence supporting the role of NAT2 in lipid and cholesterol homeostasis

### 7.1 Acetylator genotype-dependent dyslipidemia in rats congenic for *Nat2*

In rats, *Nat2* acetylator status has been shown to influence plasma lipid and cholesterol levels. In our previous study (Hong et al., 2020), we investigated the interaction between diet (control vs. high-fat) and acetylator phenotype (rapid vs. slow) using previously established congenic rat lines that exhibit rapid or slow NAT2 acetylator phenotypes. Male and female rats of each genotype were fed control or high-fat diet for 26 weeks. Regardless of the diet, rapid acetylator rats (with higher NAT2 activity) were more prone to develop dyslipidemia overall. Rapid rats exhibited higher plasma levels of triglyceride and LDL, and lower HDL level, compared to slow acetylator rats (Hong et al., 2020). Rapid acetylator rats also displayed a significantly higher total cholesterol-to-HDL ratio (Hong et al., 2020). Notably, the total cholesterol-to-HDL ratio serves as important indicators of cardiovascular risk (Millán et al., 2009). These findings suggest that rats with higher, systemic NAT2 activity exhibit dyslipidemia which raises the risk of cardiovascular dysfunction. Although there are functional differences between rat NAT2 and human NAT2 (Hein et al., 2008; Hein, 2009), this study supports the idea that high NAT2 activity results in an elevated plasma triglyceride and LDL. Furthermore, this is consistent with the aforementioned finding that the G allele of rs1495741, which is a predictor of rapid acetylator genotype (Figure 2), is associated with elevated plasma lipid and cholesterol levels (Table 1; Supplementary Table S1).

### 7.2 Transcriptional regulation of human *NAT2* by glucose and insulin

Multiple GWAS reports link non-coding *NAT2* variants to differential plasma lipid and cholesterol levels (Table 1; Supplementary Table S1). However, until recently, no non-GWAS studies have implicated human NAT2 in the process of lipid or cholesterol homeostasis. Although no direct evidence is available, our recent findings support the novel hypothesis that NAT2 is involved in regulation of lipid or cholesterol homeostasis. In HepG2 and Hep3B hepatocellular carcinoma cell lines, we observed that the transcript levels of human *NAT2* varies dynamically, depending on the nutrient status of the culture media (Hong et al., 2022). Particularly, *NAT2* transcripts are significantly upregulated by glucose (Hong et al., 2022). Glucose promotes *de novo* lipid synthesis *via* activation of ChREBP (MLXIPL) (Ortega-Prieto and Postic, 2019) or indirectly *via* insulin-SREBF1 (sterol regulatory element-binding protein 1) (Eberlé et al., 2004). In response to glucose and its metabolites (e.g., glucose-6-phosphate), ChREBP is activated and binds to a cis-acting regulatory element called “carbohydrate response element” and transactivates the target genes (Ortega-Prieto and Postic, 2019). Its target genes encode key enzymes of *de novo* lipogenesis, such as fatty acid synthase (FAS; FASN), acetyl-CoA carboxylase (ACC; ACACA) and stearoyl-CoA desaturase (SCD) (Kawaguchi et al., 2001). It is possible that NAT2 is regulated in a similar mechanism by glucose.

*NAT2* is also upregulated by insulin in HepG2 cells (Hong et al., 2022), indicating that *NAT2* may be a novel insulin-receptor target gene in hepatocytes. Elevated blood glucose induces secretion of insulin which suppresses gluconeogenesis and promotes lipogenesis in the liver. In support of this finding, a recent study reported that insulin induces mouse *Nat1* (functional homolog of human *NAT2*) expression in a mouse endothelial cancer cell line (Zou et al., 2020). In addition, cerebro-microvessels isolated from endothelial cell-specific insulin receptor knockout mice express reduced levels of both mRNA and protein of mouse *Nat1*, suggesting that insulin regulates expression of mouse *Nat1* in endothelial cells *in vivo* (Zou et al., 2020). These previous findings collectively suggest that expression of human *NAT2* (and mouse *Nat1*) is regulated by insulin in multiple cell types, including hepatocytes and endothelial cells. Importantly, this also implicates NAT2 in the process of lipogenesis, which is regulated by insulin. SREBF1 mediates the induction of lipogenic genes by insulin in hepatocytes (Horton et al., 2002). Activation of SREBF1 by insulin promotes fatty acid and cholesterol biosynthesis, for its target genes include the rate-limiting lipogenic and cholesterol biosynthetic genes, such as fatty acid synthase (*FASN*; *FAS*), acetyl-CoA carboxylase (*ACC1*; *ACACA*), HMG-CoA reductase (*HMGCR*) and the LDL receptor (*LDLR*) (Eberlé et al., 2004; Osborne and Espenshade, 2009). Transcriptional regulation of human *NAT2* by both glucose and insulin suggests that hepatic *NAT2* expression is induced by nutrient excess which coincide with the conditions that promote lipogenesis and cholesterol biosynthesis.

#### 7.2.1 Genes co-expressed with human *NAT2*

Another piece of evidence that implicates NAT2 in lipid or cholesterol homeostasis comes from an *in silico* analysis of genes co-regulated or co-expressed with human *NAT2* (Hong et al., 2022). As expected, biological processes related to xenobiotic metabolism are enriched among the co-expressed genes. Interestingly, Gene Ontology (GO) terms that are related to triglyceride, lipid, lipoprotein, and cholesterol synthesis and transport are overwhelmingly enriched among the genes co-expressed with human *NAT2*. These included “cholesterol homeostasis” (GO:0042632), “lipid homeostasis” (GO:0055088), “triglyceride homeostasis” (GO:0070328), “regulation of cholesterol transport” (GO:0032374), “plasma lipoprotein particle assembly” (GO:0034377), and “plasma lipoprotein particle remodeling” (GO:0034369) (Hong et al., 2022). Co-expressed genes, such as *APOA5*, *APOB*, *APOC2*, *APOC3*, *ABCG8*, *ANGPTL3*, *FABP1*, *MOGAT2*, and *PLA2G12B*, contributed to the enrichment of these biological processes. The findings of the *in silico* analysis strongly suggest that NAT2 plays a novel role in lipid and cholesterol metabolism and/or transport in the liver where it is mostly abundantly expressed.

The list of genes co-expressed with human *NAT2* (Hong et al., 2022) contrasts to the genes co-regulated with mouse *Nat1* (functional homolog of human *NAT2*) in adipose tissue (Chennamsetty et al., 2016). The authors reported that genes that are positively correlated with mouse *Nat1* in fat tissue are significantly enriched for biological processes associated with mitochondrial biology, glucose metabolism, and energy balance (Chennamsetty et al., 2016). The discrepancy between the two studies can be attributed to the differences between human *NAT2* and mouse *Nat1*. For example, human *NAT2* is predominantly expressed in the liver and small and large intestines and absent from adipose tissue (Single cell type - NAT2 - The Human Protein Atlas, 2022), whereas expression of mouse *Nat1* is more wide spread and present in multiple tissues (Loehle et al., 2006; Nat1 MGI Mouse Gene Detail - MGI:97279 - N-acetyl transferase 1). In addition, Laurieri and others reported that the substrate profiles of mouse NAT1 and human NAT2 are less similar than previously believed (Laurieri et al., 2014). This indicates that they may not share identical cellular functions and that findings in mouse models may not be directly applicable to humans.

## 8 Summary and conclusion

Numerous GWAS reports link non-coding human *NAT2* genetic variants to differential plasma lipid and cholesterol levels, as well as cardiometabolic disorders. A seven-intergenic-variant haplotype is associated with differential risks of dyslipidemia. Moreover, the risk alleles for dyslipidemia (e.g., rs1495741-G) are associated with rapid NAT2 acetylator phenotype, suggesting that increased systemic NAT2 activity contributes to increases in plasma lipid and cholesterol levels. The findings described herein collectively suggest that non-coding *NAT2* genetic variants play functionally important roles in regulation of lipid and cholesterol homeostasis. We propose that human *NAT2* represents a novel genetic factor that influences plasma lipid and cholesterol levels and, ultimately, alters the risk of cardiometabolic disorders. The mechanism, however, is unknown. The question of how non-coding, intergenic variants alter *NAT2* expression or activity is of high interest and subject to future studies.
