# Supplementary material for: Non-coding and intergenic genetic variants of human arylamine N-acetyltransferase 2 (NAT2) gene are associated with differential plasma lipid and cholesterol levels and cardiometabolic disorders
Source: Front Pharmacol. 2023 Apr 3;14:1091976. doi: 10.3389/fphar.2023.1091976 (PMC10106703; doi:10.3389/fphar.2023.1091976)
Supplement: Supplementary file 1 [file Table1.docx]

**Supplemental Table 1. *NAT2* genetic variants associated with differential plasma lipid, cholesterol, or fatty acid levels (Complete list)**

| **Variant** | **Type** | **Risk Allele** | **P-value** | **RAF*** | **Increase/**  **decrease** | **Reported trait** | **Reference** | **Location**† |
| --- | --- | --- | --- | --- | --- | --- | --- | --- |
| rs1115784 | Non-coding  (intronic) | G | 8 x 10^-7^ | 0.40 | NA | Myocardial infarction | (Hartiala et al., 2021) | 8:18397900 |
| rs11780610 | Non-coding | C | 9 x 10^-10^ | 0.38 | ↑ | Apolipoprotein A1 levels | (Richardson et al., 2020) | 8:18402366 |
|  |  | T | 2 x 10^-35^ | 0.62 | ↓ | Triglycerides | (Klarin et al., 2018) |  |
|  |  | T | 2 x 10^-14^ |  | NA | Mean corpuscular hemoglobin | (Chen et al., 2020) |  |
| rs11780884 | Non-coding | A | 2 x 10^-8^ | 0.32 | ↑ | Free cholesterol levels in small HDL | (Richardson et al., 2022) | 8:18388543 |
| rs11782802 | Non-coding  (intronic) | T | 2 x 10^-14^ | 0.05 | ↑ | Triglyceride levels | (Sinnott-Armstrong et al., 2021) | 8:18399145 |
| rs1208 | Coding | A | 3 x 10^-6^ | 0.68 | ↑ | Insulin resistance | (Knowles et al., 2015) | 8:18400806 |
| rs13277394 | Non-coding | T | 9 x 10^-10^ | 0.01 | ↑ | Triglyceride levels | (Sinnott-Armstrong et al., 2021) | 8:18408709 |
|  |  | T | 9 x 10^-11^ | 0.01 | ↑ | Total cholesterol levels | (Sinnott-Armstrong et al., 2021) |  |
| rs146812806 | Non-coding | Ins | 9 x 10^-14^ | NR | ↓ | Mean corpuscular hemoglobin concentration | (Chen et al., 2020) | 8:18414994 |
| rs1495741 | Non-coding | NR | 3 x 10^-28^ | - | ↑ | Low density lipoprotein cholesterol levels | (Ripatti et al., 2020) | 8:18415371 |
|  |  | A | 4 x 10^-8^ | 0.65 | ↓ | Triglyceride levels | (Bentley et al., 2019) |  |
|  |  | A | 1 x 10^-27^ |  | ↑ | Blood metabolite levels (1-methylurate) | (Shin et al., 2014) |  |
|  |  | A | 1 x 10^-19^ |  | ↓ | Medication use (HMG-CoA reductase inhibitors) | (Sakaue et al., 2021) |  |
|  |  | G | 4 x 10^-14^ | 0.35 | ↑ | Triglycerides | (Teslovich et al., 2010) |  |
|  |  | G | 2 x 10^-9^ |  | ↑ | Cholesterol, total | (Teslovich et al., 2010) |  |
|  |  | G | 7 x 10^-56^ |  | ↑ | Triglyceride levels | (Richardson et al., 2020) |  |
|  |  | G | 5 x 10^-12^ |  | ↑ | LDL cholesterol levels | (Richardson et al., 2020) |  |
|  |  | G | 6 x 10^-6^ |  | ↑ | Low density lipoprotein cholesterol levels | (Hoffmann et al., 2018) |  |
|  |  | G | 3 x 10^-8^ |  | ↑ | Cholesterol, total | (Willer et al., 2013) |  |
|  |  | G | 3 x 10^-12^ |  | ↑ | Triglycerides | (Willer et al., 2013) |  |
|  |  | G | 7 x 10^-16^ |  | ↑ | Triglycerides | (Hoffmann et al., 2018) |  |
|  |  | G | 3 x 10^-6^ |  | ↑ | Triglycerides | (Hoffmann et al., 2018) |  |
|  |  | G | 6 x 10^-22^ |  | ↑ | Triglycerides | (Hoffmann et al., 2018) |  |
|  |  | G | 4 x 10^-16^ |  | ↑ | Total cholesterol levels | (Hoffmann et al., 2018) |  |
|  |  | G | 1 x 10^-13^ |  | ↑ | Total cholesterol levels | (Hoffmann et al., 2018) |  |
|  |  | G | 4 x 10^-20^ |  | ↑ | Triglyceride levels in large HDL | (Richardson et al., 2022) |  |
|  |  | G | 3 x 10^-10^ |  | ↑ | Phospholipids to total lipids ratio in large VLDL | (Richardson et al., 2022) |  |
|  |  | G | 3 x 10^-18^ |  | ↓ | Cholesteryl esters to total lipids ratio in medium HDL | (Richardson et al., 2022) |  |
|  |  | G | 8 x 10^-16^ |  | ↓ | Cholesterol to total lipids ratio in medium HDL | (Richardson et al., 2022) |  |
|  |  | G | 4 x 10^-12^ |  | ↑ | Triglyceride levels in LDL | (Richardson et al., 2022) |  |
|  |  | G | 1 x 10^-19^ |  | ↑ | Triglycerides to total lipids ratio in medium HDL | (Richardson et al., 2022) |  |
|  |  | G | 3 x 10^-9^ |  | ↑ | Triglycerides to total lipids ratio in medium LDL | (Richardson et al., 2022) |  |
|  |  | G | 9 x 10^-29^ |  | ↑ | Triglyceride levels in medium HDL | (Richardson et al., 2022) |  |
|  |  | G | 4 x 10^-18^ |  | ↑ | Monounsaturated fatty acid levels | (Richardson et al., 2022) |  |
|  |  | G | 1 x 10^-8^ |  | ↑ | Phospholipids to total lipids ratio in medium HDL | (Richardson et al., 2022) |  |
|  |  | G | 2 x 10^-12^ |  | ↑ | Triglyceride levels in medium LDL | (Richardson et al., 2022) |  |
|  |  | G | 2 x 10^-16^ |  | ↓ | Free cholesterol to total lipids ratio in medium LDL | (Richardson et al., 2022) |  |
|  |  | G | 5 x 10^-12^ |  | ↓ | Ratio of polyunsaturated fatty acids to total fatty acids | (Richardson et al., 2022) |  |
|  |  | G | 4 x 10^-20^ |  | ↓ | Free cholesterol to total lipids ratio in small LDL | (Richardson et al., 2022) |  |
|  |  | G | 1 x 10^-16^ |  | ↓ | Phospholipids to total lipids ratio in small LDL | (Richardson et al., 2022) |  |
|  |  | G | 2 x 10^-21^ |  | ↓ | Cholesterol to total lipids ratio in small HDL | (Richardson et al., 2022) |  |
|  |  | G | 4 x 10^-15^ |  | ↑ | Ratio of monounsaturated fatty acids to total fatty acids | (Richardson et al., 2022) |  |
|  |  | G | 1 x 10^-12^ |  | ↓ | Ratio of omega-6 fatty acids to total fatty acids | (Richardson et al., 2022) |  |
|  |  | G | 2 x 10^-14^ |  | ↓ | Ratio of polyunsaturated fatty acids to monounsaturated fatty acids | (Richardson et al., 2022) |  |
|  |  | G | 3 x 10^-18^ |  | ↓ | Cholesteryl esters to total lipids ratio in small HDL | (Richardson et al., 2022) |  |
|  |  | G | 7 x 10^-10^ |  | ↑ | Phospholipids to total lipids ratio in small HDL | (Richardson et al., 2022) |  |
|  |  | G | 1 x 10^-15^ |  | ↑ | Triglycerides to total lipids ratio in small HDL | (Richardson et al., 2022) |  |
|  |  | G | 1 x 10^-16^ |  | ↑ | Cholesteryl esters to total lipids ratio in small LDL | (Richardson et al., 2022) |  |
|  |  | G | 1 x 10^-20^ |  | ↑ | Triglycerides to total lipids ratio in large HDL | (Richardson et al., 2022) |  |
|  |  | G | 1 x 10^-10^ |  | ↑ | Phospholipids to total lipids ratio in large HDL | (Richardson et al., 2022) |  |
|  |  | G | 7 x 10^-15^ |  | ↑ | Triglyceride levels in small LDL | (Richardson et al., 2022) |  |
|  |  | G | 6 x 10^-17^ |  | ↑ | Triglycerides to total lipids ratio in small LDL | (Richardson et al., 2022) |  |
|  |  | G | 8 x 10^-14^ |  | ↑ | Saturated fatty acid levels | (Richardson et al., 2022) |  |
|  |  | G | 9 x 10^-15^ |  | ↑ | Total fatty acid levels | (Richardson et al., 2022) |  |
|  |  | G | 8 x 10^-19^ |  | ↑ | Triglycerides to total lipids ratio in very large HDL | (Richardson et al., 2022) |  |
|  |  | G | 2 x 10^-15^ |  | ↑ | Concentration of chylomicrons and extremely large VLDL particles | (Richardson et al., 2022) |  |
|  |  | G | 3 x 10^-15^ |  | ↑ | Triglyceride levels in very small VLDL | (Richardson et al., 2022) |  |
|  |  | G | 2 x 10^-16^ |  | ↑ | Cholesterol levels in chylomicrons and extremely large VLDL | (Richardson et al., 2022) |  |
|  |  | G | 6 x 10^-17^ |  | ↑ | Free cholesterol levels in chylomicrons and extremely large VLDL | (Richardson et al., 2022) |  |
|  |  | G | 6 x 10^-14^ |  | ↑ | Triglyceride levels in very large HDL | (Richardson et al., 2022) |  |
|  |  | G | 3 x 10^-14^ |  | ↑ | Total lipid levels in chylomicrons and extremely large VLDL | (Richardson et al., 2022) |  |
|  |  | G | 9 x 10^-11^ |  | ↓ | Cholesteryl esters to total lipids ratio in very large HDL | (Richardson et al., 2022) |  |
|  |  | G | 2 x 10^-12^ |  | ↑ | Triglyceride levels in IDL | (Richardson et al., 2022) |  |
|  |  | G | 1 x 10^-17^ |  | ↑ | Medication use (HMG-CoA reductase inhibitors) | (Wu et al., 2019) |  |
|  |  | G | 2 x 10^-27^ |  | ↑ | Triglyceride levels in HDL | (Richardson et al., 2022) |  |
|  |  | G | 5 x 10^-21^ |  | ↓ | Cholesteryl esters to total lipids ratio in large HDL | (Richardson et al., 2022) |  |
|  |  | G | 4 x 10^-10^ |  | ↑ | Apolipoprotein B levels | (Richardson et al., 2020) |  |
|  |  | G | 6 x 10^-11^ |  | ↑ | Triglyceride levels in large LDL | (Richardson et al., 2022) |  |
|  |  | G | 2 x 10^-13^ |  | ↓ | Free cholesterol to total lipids ratio in large LDL | (Richardson et al., 2022) |  |
|  |  | G | 7 x 10^-16^ |  | ↑ | Cholesteryl ester levels in chylomicrons and extremely large VLDL | (Richardson et al., 2022) |  |
| rs1495743 | Non-coding | NR | 1 x 10^-8^ | - | ↓ | Triglyceride levels | (Kanai et al., 2018) | 8:18415790 |
|  |  | NR | 4 x 10^-14^ | - | ↑ | Triglyceride levels | (Nielsen et al., 2020) |  |
|  |  | C | 1 x 10^-19^ | 0.65 | ↓ | Triglyceride levels in current drinkers | (de Vries et al., 2019) |  |
|  |  | C | 1 x 10^-10^ |  | ↓ | Triglyceride levels in current drinkers | (de Vries et al., 2019) |  |
|  |  | C | 3 x 10^-29^ |  | ↓ | Triglyceride levels x alcohol consumption (drinkers vs non-drinkers) | (de Vries et al., 2019) |  |
|  |  | C | 3 x 10^-8^ |  | ↓ | LDL cholesterol levels x alcohol consumption (drinkers vs non-drinkers) | (de Vries et al., 2019) |  |
|  |  | C | 2 x 10^-7^ |  | ↓ | Triglyceride levels | (de Vries et al., 2019) |  |
|  |  | C | 5 x 10^-9^ |  | ↓ | Triglyceride levels | (de Vries et al., 2019) |  |
|  |  | C | 8 x 10^-7^ |  | ↓ | LDL cholesterol levels x alcohol consumption (regular vs non-regular) | (de Vries et al., 2019) |  |
|  |  | C | 8 x 10^-12^ |  | ↓ | Triglyceride levels x alcohol consumption (drinkers vs non-drinkers) | (de Vries et al., 2019) |  |
|  |  | C | 4 x 10^-9^ |  | ↓ | Triglyceride levels in current drinkers | (de Vries et al., 2019) |  |
|  |  | C | 1 x 10^-16^ |  | ↓ | Triglyceride levels x alcohol consumption (drinkers vs non-drinkers) | (de Vries et al., 2019) |  |
|  |  | C | 9 x 10^-8^ |  | ↓ | LDL cholesterol levels x alcohol consumption (drinkers vs non-drinkers) | (de Vries et al., 2019) |  |
|  |  | C | 7 x 10^-15^ |  | ↓ | Triglyceride levels | (de Vries et al., 2019) |  |
|  |  | C | 3 x 10^-16^ |  | ↓ | Triglyceride levels x alcohol consumption (regular vs non-regular) | (de Vries et al., 2019) |  |
|  |  | C | 3 x 10^-7^ |  | ↓ | LDL cholesterol levels x alcohol consumption (regular vs non-regular) | (de Vries et al., 2019) |  |
|  |  | C | 2 x 10^-11^ |  | ↓ | Triglyceride levels x alcohol consumption (regular vs non-regular) | (de Vries et al., 2019) |  |
|  |  | C | 2 x 10^-28^ |  | ↓ | Triglyceride levels x alcohol consumption (regular vs non-regular) | (de Vries et al., 2019) |  |
|  |  | C | 7 x 10^-11^ |  | ↓ | LDL cholesterol | (Sakaue et al., 2021) |  |
|  |  | G | 3 x 10^-6^ | 0.35 | ↑ | Low density lipoprotein cholesterol levels | (Hoffmann et al., 2018) |  |
|  |  | G | 5 x 10^-16^ |  | ↑ | Triglycerides | (Hoffmann et al., 2018) |  |
|  |  | G | 1 x 10^-6^ |  | ↑ | Triglycerides | (Hoffmann et al., 2018) |  |
|  |  | G | 3 x 10^-22^ |  | ↑ | Triglycerides | (Hoffmann et al., 2018) |  |
|  |  | G | 3 x 10^-14^ |  | ↑ | Total cholesterol levels | (Hoffmann et al., 2018) |  |
|  |  | G | 8 x 10^-17^ |  | ↑ | Total cholesterol levels | (Hoffmann et al., 2018) |  |
| rs1495745 | Non-coding | T | 9 x 10^-9^ | 0.38 | ↓ | Cholesteryl esters to total lipids ratio in very small VLDL | (Richardson et al., 2022) | 8:18405213 |
| rs1961456 | Non-coding  (intronic) | A | 7 x 10^-17^ | 0.59 | ↓ | Total cholesterol levels | (Spracklen et al., 2017) | 8:18398199 |
| rs34987019 | Non-coding | T | 2 x 10^-8^ | 0.73 | ↓ | Total cholesterol levels | (Klarin et al., 2018) | 8:18416933 |
| rs35246381 | Non-coding | NR | 3 x 10^-13^ | - | ↓ | Triglyceride levels x short total sleep time interaction (2df test) | (Noordam et al., 2019) | 8:18415025 |
|  |  | C | 2 x 10^-13^ | 0.35 | ↓ | Cholesteryl esters to total lipids ratio in medium VLDL | (Richardson et al., 2022) |  |
|  |  | C | 5 x 10^-9^ |  | ↓ | Free cholesterol to total lipids ratio in medium VLDL | (Richardson et al., 2022) |  |
|  |  | C | 2 x 10^-12^ |  | ↓ | Cholesterol to total lipids ratio in medium VLDL | (Richardson et al., 2022) |  |
|  |  | C | 1 x 10^-10^ |  | ↑ | Triglycerides to total lipids ratio in medium VLDL | (Richardson et al., 2022) |  |
|  |  | C | 1 x 10^-9^ |  | ↑ | Phosphoglycerides levels | (Richardson et al., 2022) |  |
|  |  | C | 2 x 10^-8^ |  | ↑ | Phosphatidylcholine levels | (Richardson et al., 2022) |  |
|  |  | C | 4 x 10^-14^ |  | ↑ | Phospholipid levels in small HDL | (Richardson et al., 2022) |  |
|  |  | C | 1 x 10^-13^ |  | ↓ | Free cholesterol to total lipids ratio in small VLDL | (Richardson et al., 2022) |  |
|  |  | C | 4 x 10^-10^ |  | ↓ | Cholesterol to total lipids ratio in small VLDL | (Richardson et al., 2022) |  |
|  |  | C | 2 x 10^-13^ |  | ↓ | Phospholipids to total lipids ratio in small VLDL | (Richardson et al., 2022) |  |
|  |  | C | 2 x 10^-11^ |  | ↑ | Triglycerides to total lipids ratio in small VLDL | (Richardson et al., 2022) |  |
|  |  | C | 1 x 10^-9^ |  | ↓ | Cholesterol to total lipids ratio in very small VLDL | (Richardson et al., 2022) |  |
|  |  | C | 2 x 10^-11^ |  | ↑ | Triglycerides to total lipids ratio in very small VLDL | (Richardson et al., 2022) |  |
|  |  | C | 6 x 10^-20^ |  | ↓ | Cholesterol to total lipids ratio in large HDL | (Richardson et al., 2022) |  |
|  |  | C | 2 x 10^-17^ |  | ↓ | Free cholesterol to total lipids ratio in IDL | (Richardson et al., 2022) |  |
|  |  | C | 1 x 10^-13^ |  | ↓ | Mean corpuscular hemoglobin concentration | (Vuckovic et al., 2020) |  |
|  |  | C | 2 x 10^-14^ |  | ↓ | Free cholesterol to total lipids ratio in very small VLDL | (Richardson et al., 2022) |  |
|  |  | T | 2 x 10^-18^ | 0.65 | NA | Triglycerides x physical activity interaction (2df test) | (Kilpeläinen et al., 2019) |  |
| rs35570672 | Non-coding | C | 3 x 10^-10^ | 0.65 | ↓ | Mean corpuscular hemoglobin | (Vuckovic et al., 2020) | 8:18415125 |
|  |  | C | 4 x 10^-8^ |  | ↓ | Mean corpuscular hemoglobin | (Sakaue et al., 2021) |  |
| rs35583283 | Non-coding | G | 1 x 10^-16^ | 0.71 | ↓ | Familial combined hyperlipidemia defined by Consensus criteria | (Trinder et al., 2022) | 8:18396999 |
|  |  | G | 4 x 10^-16^ |  | ↓ | Familial combined hyperlipidemia defined by Dutch criteria | (Trinder et al., 2022) |  |
| rs4646248 | Non-coding | NR | 5 x 10^-8^ | - | ↓ | Coronary artery disease | (Koyama et al., 2020) | 8:18402845 |
|  |  | C | 3 x 10^-9^ | 0.39 | ↑ | Triglycerides to total lipids ratio in large LDL | (Richardson et al., 2022) |  |
|  |  | C | 2 x 10^-9^ |  | ↑ | Triglycerides to total lipids ratio in IDL | (Richardson et al., 2022) |  |
|  |  | T | 2 x 10^-7^ | 0.61 | ↓ | Coronary artery disease | (van der Harst and Verweij, 2018) |  |
| rs4921913 | Non-coding | NR | 9 x 10^-35^ | - | ↑ | Triglyceride levels | (Ripatti et al., 2020) | 8:18414867 |
|  |  | C | 5 x 10^-8^ | 0.35 | ↑ | Metabolic syndrome | (Lind, 2019) |  |
|  |  | C | 2 x 10^-10^ |  | ↑ | Cholesterol levels in large VLDL | (Richardson et al., 2022) |  |
|  |  | C | 2 x 10^-8^ |  | ↑ | Cholesteryl ester levels in large VLDL | (Richardson et al., 2022) |  |
|  |  | C | 6 x 10^-12^ |  | ↑ | Free cholesterol levels in large VLDL | (Richardson et al., 2022) |  |
|  |  | C | 9 x 10^-12^ |  | ↑ | Total lipid levels in large VLDL | (Richardson et al., 2022) |  |
|  |  | C | 3 x 10^-11^ |  | ↑ | Triglyceride levels in large VLDL | (Richardson et al., 2022) |  |
|  |  | C | 7 x 10^-13^ |  | ↑ | Phospholipid levels in large VLDL | (Richardson et al., 2022) |  |
|  |  | C | 2 x 10^-12^ |  | ↑ | Concentration of large VLDL particles | (Richardson et al., 2022) |  |
|  |  | C | 3 x 10^-12^ |  | ↑ | Triglyceride levels in medium VLDL | (Richardson et al., 2022) |  |
|  |  | C | 2 x 10^-8^ |  | ↑ | Omega-6 fatty acid levels | (Richardson et al., 2022) |  |
|  |  | C | 2 x 10^-10^ |  | ↑ | Phospholipid levels in VLDL | (Richardson et al., 2022) |  |
|  |  | C | 3 x 10^-12^ |  | ↑ | Average diameter for VLDL particles | (Richardson et al., 2022) |  |
|  |  | C | 1 x 10^-14^ |  | ↑ | Triglyceride levels in VLDL | (Richardson et al., 2022) |  |
|  |  | C | 2 x 10^-9^ |  | ↑ | Concentration of small VLDL particles | (Richardson et al., 2022) |  |
|  |  | C | 2 x 10^-9^ |  | ↑ | Total lipid levels in small VLDL | (Richardson et al., 2022) |  |
|  |  | C | 2 x 10^-8^ |  | ↑ | Free cholesterol levels in VLDL | (Richardson et al., 2022) |  |
|  |  | C | 5 x 10^-12^ |  | ↑ | Total lipid levels in VLDL | (Richardson et al., 2022) |  |
|  |  | C | 3 x 10^-13^ |  | ↑ | Phospholipid levels in very large VLDL | (Richardson et al., 2022) |  |
|  |  | C | 1 x 10^-12^ |  | ↑ | Triglyceride levels in chylomicrons and extremely large VLDL | (Richardson et al., 2022) |  |
|  |  | C | 5 x 10^-14^ |  | ↑ | Concentration of very large VLDL particles | (Richardson et al., 2022) |  |
|  |  | C | 4 x 10^-11^ |  | ↑ | Cholesterol levels in very large VLDL | (Richardson et al., 2022) |  |
|  |  | C | 7 x 10^-14^ |  | ↑ | Triglyceride levels in very large VLDL | (Richardson et al., 2022) |  |
|  |  | C | 6 x 10^-9^ |  | ↑ | Cholesteryl ester levels in very large VLDL | (Richardson et al., 2022) |  |
|  |  | C | 1 x 10^-13^ |  | ↑ | Total lipid levels in very large VLDL | (Richardson et al., 2022) |  |
| rs4921914 | Non-coding | NR | 7 x 10^-12^ | - | ↑ | Total cholesterol levels | (Nielsen et al., 2020) | 8:18414928 |
|  |  | T | 7 x 10^-8^ | 0.65 | ↓ | Triglyceride levels | (Bentley et al., 2019) |  |
|  |  | T | 9 x 10^-12^ |  | NA | Mean corpuscular hemoglobin concentration | (Chen et al., 2020) |  |
|  |  | T | 2 x 10^-20^ |  | ↓ | Triglyceride levels | (Spracklen et al., 2017) |  |
| rs4921915 | Non-coding | A | 2 x 10^-10^ | 0.65 | ↓ | Triglycerides | (Sakaue et al., 2021) | 8:18414956 |
|  |  | A | 3 x 10^-22^ |  | ↓ | Total cholesterol levels | (Sakaue et al., 2021) |  |
|  |  | G | 1 x 10^-11^ | 0.35 | ↑ | Total lipid levels in small HDL | (Richardson et al., 2022) |  |
|  |  | G | 9 x 10^-22^ |  | ↑ | Triglyceride levels in small HDL | (Richardson et al., 2022) |  |
|  |  | G | 4 x 10^-16^ |  | ↑ | Triglyceride levels in small VLDL | (Richardson et al., 2022) |  |
|  |  | G | 2 x 10^-16^ |  | ↑ | Total triglycerides levels | (Richardson et al., 2022) |  |
|  |  | G | 4 x 10^-11^ |  | ↑ | Ratio of triglycerides to phosphoglycerides | (Richardson et al., 2022) |  |
|  |  | G | 6 x 10^-13^ |  | ↑ | Free cholesterol levels in very large VLDL | (Richardson et al., 2022) |  |
|  |  | G | 6 x 10^-10^ |  | ↓ | Cholesterol to total lipids ratio in very large VLDL | (Richardson et al., 2022) |  |
|  |  | G | 5 x 10^-17^ |  | ↑ | Phospholipid levels in chylomicrons and extremely large VLDL | (Richardson et al., 2022) |  |
|  |  | G | 1 x 10^-10^ |  | ↓ | Cholesteryl esters to total lipids ratio in very large VLDL | (Richardson et al., 2022) |  |
|  |  | G | 2 x 10^-8^ |  | ↑ | Polyunsaturated fatty acid levels | (Richardson et al., 2022) |  |
| rs6997340 | Non-coding | T | 5 x 10^-9^ | 0.43 | ↑ | Coronary artery disease | (van der Harst and Verweij, 2018) | 8:18429487 |
| rs721399 | Non-coding | T | 9 x 10^-13^ | 0.56 | ↓ | Mean corpuscular hemoglobin | (Chen et al., 2020) | 8:18401856 |
| rs73207888 | Non-coding | T | 3 x 10^-8^ | 0.14 | ↑ | Medication use (calcium channel blockers) | (Wu et al., 2019) | 8:18425421 |

*, based on 1000 Genomes Project (phase 3)

†, Human GRCh38/hg38

RAF, relative allele frequency. NR, not reported. NA, not available.

Ins, insertion

**References**

Avery, C. L., Sitlani, C. M., Arking, D. E., Arnett, D. K., Bis, J. C., Boerwinkle, E., et al. (2014). Drug-gene interactions and the search for missing heritability: a cross-sectional pharmacogenomics study of the QT interval. *Pharmacogenomics J.* 14, 6–13. doi: 10.1038/tpj.2013.4.

Bar, N., Korem, T., Weissbrod, O., Zeevi, D., Rothschild, D., Leviatan, S., et al. (2020). A reference map of potential determinants for the human serum metabolome. *Nature* 588, 135–140. doi: 10.1038/s41586-020-2896-2.

Bell, S., Rigas, A. S., Magnusson, M. K., Ferkingstad, E., Allara, E., Bjornsdottir, G., et al. (2021). A genome-wide meta-analysis yields 46 new loci associating with biomarkers of iron homeostasis. *Commun. Biol.* 4, 156. doi: 10.1038/s42003-020-01575-z.

Bentley, A. R., Sung, Y. J., Brown, M. R., Winkler, T. W., Kraja, A. T., Ntalla, I., et al. (2019). Multi-ancestry genome-wide gene-smoking interaction study of 387,272 individuals identifies new loci associated with serum lipids. *Nat. Genet.* 51, 636–648. doi: 10.1038/s41588-019-0378-y.

Benyamin, B., Esko, T., Ried, J. S., Radhakrishnan, A., Vermeulen, S. H., Traglia, M., et al. (2014). Novel loci affecting iron homeostasis and their effects in individuals at risk for hemochromatosis. *Nat. Commun.* 5, 4926. doi: 10.1038/ncomms5926.

Chen, M.-H., Raffield, L. M., Mousas, A., Sakaue, S., Huffman, J. E., Moscati, A., et al. (2020). Trans-ethnic and Ancestry-Specific Blood-Cell Genetics in 746,667 Individuals from 5 Global Populations. *Cell* 182, 1198-1213.e14. doi: 10.1016/j.cell.2020.06.045.

de Vries, P. S., Brown, M. R., Bentley, A. R., Sung, Y. J., Winkler, T. W., Ntalla, I., et al. (2019). Multiancestry Genome-Wide Association Study of Lipid Levels Incorporating Gene-Alcohol Interactions. *Am. J. Epidemiol.* 188, 1033–1054. doi: 10.1093/aje/kwz005.

Donati, G., Dumontheil, I., Pain, O., Asbury, K., and Meaburn, E. L. (2021). Evidence for specificity of polygenic contributions to attainment in English, maths and science during adolescence. *Sci. Rep.* 11, 3851. doi: 10.1038/s41598-021-82877-y.

Feofanova, E. V., Chen, H., Dai, Y., Jia, P., Grove, M. L., Morrison, A. C., et al. (2020). A Genome-wide Association Study Discovers 46 Loci of the Human Metabolome in the Hispanic Community Health Study/Study of Latinos. *Am. J. Hum. Genet.* 107, 849–863. doi: 10.1016/j.ajhg.2020.09.003.

Figueroa, J. D., Ye, Y., Siddiq, A., Garcia-Closas, M., Chatterjee, N., Prokunina-Olsson, L., et al. (2014). Genome-wide association study identifies multiple loci associated with bladder cancer risk. *Hum. Mol. Genet.* 23, 1387–1398. doi: 10.1093/hmg/ddt519.

Han, X., Ong, J.-S., An, J., Hewitt, A. W., Gharahkhani, P., and MacGregor, S. (2020). Using Mendelian randomization to evaluate the causal relationship between serum C-reactive protein levels and age-related macular degeneration. *Eur. J. Epidemiol.* 35, 139–146. doi: 10.1007/s10654-019-00598-z.

Hartiala, J. A., Han, Y., Jia, Q., Hilser, J. R., Huang, P., Gukasyan, J., et al. (2021). Genome-wide analysis identifies novel susceptibility loci for myocardial infarction. *Eur. Heart J.* 42, 919–933. doi: 10.1093/eurheartj/ehaa1040.

Hoffmann, T. J., Theusch, E., Haldar, T., Ranatunga, D. K., Jorgenson, E., Medina, M. W., et al. (2018). A large electronic-health-record-based genome-wide study of serum lipids. *Nat. Genet.* 50, 401–413. doi: 10.1038/s41588-018-0064-5.

Kanai, M., Akiyama, M., Takahashi, A., Matoba, N., Momozawa, Y., Ikeda, M., et al. (2018). Genetic analysis of quantitative traits in the Japanese population links cell types to complex human diseases. *Nat. Genet.* 50, 390–400. doi: 10.1038/s41588-018-0047-6.

Kilpeläinen, T. O., Bentley, A. R., Noordam, R., Sung, Y. J., Schwander, K., Winkler, T. W., et al. (2019). Multi-ancestry study of blood lipid levels identifies four loci interacting with physical activity. *Nat. Commun.* 10, 376. doi: 10.1038/s41467-018-08008-w.

Klarin, D., Damrauer, S. M., Cho, K., Sun, Y. V., Teslovich, T. M., Honerlaw, J., et al. (2018). Genetics of blood lipids among ~300,000 multi-ethnic participants of the Million Veteran Program. *Nat. Genet.* 50, 1514–1523. doi: 10.1038/s41588-018-0222-9.

Knowles, J. W., Xie, W., Zhang, Z., Chennamsetty, I., Chennemsetty, I., Assimes, T. L., et al. (2015). Identification and validation of N-acetyltransferase 2 as an insulin sensitivity gene. *J. Clin. Invest.* 125, 1739–1751. doi: 10.1172/JCI74692.

Koyama, S., Ito, K., Terao, C., Akiyama, M., Horikoshi, M., Momozawa, Y., et al. (2020). Population-specific and trans-ancestry genome-wide analyses identify distinct and shared genetic risk loci for coronary artery disease. *Nat. Genet.* 52, 1169–1177. doi: 10.1038/s41588-020-0705-3.

Krumsiek, J., Suhre, K., Evans, A. M., Mitchell, M. W., Mohney, R. P., Milburn, M. V., et al. (2012). Mining the unknown: a systems approach to metabolite identification combining genetic and metabolic information. *PLoS Genet.* 8, e1003005. doi: 10.1371/journal.pgen.1003005.

Lind, L. (2019). Genome-Wide Association Study of the Metabolic Syndrome in UK Biobank. *Metab. Syndr. Relat. Disord.* 17, 505–511. doi: 10.1089/met.2019.0070.

Müller, S. H., Girard, S. L., Hopfner, F., Merner, N. D., Bourassa, C. V., Lorenz, D., et al. (2016). Genome-wide association study in essential tremor identifies three new loci. *Brain J. Neurol.* 139, 3163–3169. doi: 10.1093/brain/aww242.

Nielsen, J. B., Rom, O., Surakka, I., Graham, S. E., Zhou, W., Roychowdhury, T., et al. (2020). Loss-of-function genomic variants highlight potential therapeutic targets for cardiovascular disease. *Nat. Commun.* 11, 6417. doi: 10.1038/s41467-020-20086-3.

Noordam, R., Bos, M. M., Wang, H., Winkler, T. W., Bentley, A. R., Kilpeläinen, T. O., et al. (2019). Multi-ancestry sleep-by-SNP interaction analysis in 126,926 individuals reveals lipid loci stratified by sleep duration. *Nat. Commun.* 10, 5121. doi: 10.1038/s41467-019-12958-0.

Pazoki, R., Vujkovic, M., Elliott, J., Evangelou, E., Gill, D., Ghanbari, M., et al. (2021). Genetic analysis in European ancestry individuals identifies 517 loci associated with liver enzymes. *Nat. Commun.* 12, 2579. doi: 10.1038/s41467-021-22338-2.

Raffler, J., Friedrich, N., Arnold, M., Kacprowski, T., Rueedi, R., Altmaier, E., et al. (2015). Genome-Wide Association Study with Targeted and Non-targeted NMR Metabolomics Identifies 15 Novel Loci of Urinary Human Metabolic Individuality. *PLoS Genet.* 11, e1005487. doi: 10.1371/journal.pgen.1005487.

Rhee, E. P., Surapaneni, A., Zheng, Z., Zhou, L., Dutta, D., Arking, D. E., et al. (2022). Trans-ethnic genome-wide association study of blood metabolites in the Chronic Renal Insufficiency Cohort (CRIC) study. *Kidney Int.* 101, 814–823. doi: 10.1016/j.kint.2022.01.014.

Richardson, T. G., Leyden, G. M., Wang, Q., Bell, J. A., Elsworth, B., Davey Smith, G., et al. (2022). Characterising metabolomic signatures of lipid-modifying therapies through drug target mendelian randomisation. *PLoS Biol.* 20, e3001547. doi: 10.1371/journal.pbio.3001547.

Richardson, T. G., Sanderson, E., Palmer, T. M., Ala-Korpela, M., Ference, B. A., Davey Smith, G., et al. (2020). Evaluating the relationship between circulating lipoprotein lipids and apolipoproteins with risk of coronary heart disease: A multivariable Mendelian randomisation analysis. *PLoS Med.* 17, e1003062. doi: 10.1371/journal.pmed.1003062.

Ripatti, P., Rämö, J. T., Mars, N. J., Fu, Y., Lin, J., Söderlund, S., et al. (2020). Polygenic Hyperlipidemias and Coronary Artery Disease Risk. *Circ. Genomic Precis. Med.* 13, e002725. doi: 10.1161/CIRCGEN.119.002725.

Roberts, V., Main, B., Timpson, N. J., and Haworth, S. (2020). Genome-Wide Association Study Identifies Genetic Associations with Perceived Age. *J. Invest. Dermatol.* 140, 2380–2385. doi: 10.1016/j.jid.2020.03.970.

Rothman, N., Garcia-Closas, M., Chatterjee, N., Malats, N., Wu, X., Figueroa, J. D., et al. (2010). A multi-stage genome-wide association study of bladder cancer identifies multiple susceptibility loci. *Nat. Genet.* 42, 978–984. doi: 10.1038/ng.687.

Rueedi, R., Ledda, M., Nicholls, A. W., Salek, R. M., Marques-Vidal, P., Morya, E., et al. (2014). Genome-wide association study of metabolic traits reveals novel gene-metabolite-disease links. *PLoS Genet.* 10, e1004132. doi: 10.1371/journal.pgen.1004132.

Sakaue, S., Kanai, M., Tanigawa, Y., Karjalainen, J., Kurki, M., Koshiba, S., et al. (2021). A cross-population atlas of genetic associations for 220 human phenotypes. *Nat. Genet.* 53, 1415–1424. doi: 10.1038/s41588-021-00931-x.

Schlosser, P., Li, Y., Sekula, P., Raffler, J., Grundner-Culemann, F., Pietzner, M., et al. (2020). Genetic studies of urinary metabolites illuminate mechanisms of detoxification and excretion in humans. *Nat. Genet.* 52, 167–176. doi: 10.1038/s41588-019-0567-8.

Shin, S.-Y., Fauman, E. B., Petersen, A.-K., Krumsiek, J., Santos, R., Huang, J., et al. (2014). An atlas of genetic influences on human blood metabolites. *Nat. Genet.* 46, 543–550. doi: 10.1038/ng.2982.

Sinnott-Armstrong, N., Tanigawa, Y., Amar, D., Mars, N., Benner, C., Aguirre, M., et al. (2021). Genetics of 35 blood and urine biomarkers in the UK Biobank. *Nat. Genet.* 53, 185–194. doi: 10.1038/s41588-020-00757-z.

Spracklen, C. N., Chen, P., Kim, Y. J., Wang, X., Cai, H., Li, S., et al. (2017). Association analyses of East Asian individuals and trans-ancestry analyses with European individuals reveal new loci associated with cholesterol and triglyceride levels. *Hum. Mol. Genet.* 26, 1770–1784. doi: 10.1093/hmg/ddx062.

Suhre, K., Shin, S.-Y., Petersen, A.-K., Mohney, R. P., Meredith, D., Wägele, B., et al. (2011a). Human metabolic individuality in biomedical and pharmaceutical research. *Nature* 477, 54–60. doi: 10.1038/nature10354.

Suhre, K., Wallaschofski, H., Raffler, J., Friedrich, N., Haring, R., Michael, K., et al. (2011b). A genome-wide association study of metabolic traits in human urine. *Nat. Genet.* 43, 565–569. doi: 10.1038/ng.837.

Suvichapanich, S., Wattanapokayakit, S., Mushiroda, T., Yanai, H., Chuchottawon, C., Kantima, T., et al. (2019). Genomewide Association Study Confirming the Association of NAT2 with Susceptibility to Antituberculosis Drug-Induced Liver Injury in Thai Patients. *Antimicrob. Agents Chemother.* 63, e02692-18. doi: 10.1128/AAC.02692-18.

Teslovich, T. M., Musunuru, K., Smith, A. V., Edmondson, A. C., Stylianou, I. M., Koseki, M., et al. (2010). Biological, clinical and population relevance of 95 loci for blood lipids. *Nature* 466, 707–713. doi: 10.1038/nature09270.

Trinder, M., Vikulova, D., Pimstone, S., Mancini, G. B. J., and Brunham, L. R. (2022). Polygenic architecture and cardiovascular risk of familial combined hyperlipidemia. *Atherosclerosis* 340, 35–43. doi: 10.1016/j.atherosclerosis.2021.11.032.

van der Harst, P., and Verweij, N. (2018). Identification of 64 Novel Genetic Loci Provides an Expanded View on the Genetic Architecture of Coronary Artery Disease. *Circ. Res.* 122, 433–443. doi: 10.1161/CIRCRESAHA.117.312086.

Vuckovic, D., Bao, E. L., Akbari, P., Lareau, C. A., Mousas, A., Jiang, T., et al. (2020). The Polygenic and Monogenic Basis of Blood Traits and Diseases. *Cell* 182, 1214-1231.e11. doi: 10.1016/j.cell.2020.08.008.

Willer, C. J., Schmidt, E. M., Sengupta, S., Peloso, G. M., Gustafsson, S., Kanoni, S., et al. (2013). Discovery and refinement of loci associated with lipid levels. *Nat. Genet.* 45, 1274–1283. doi: 10.1038/ng.2797.

Wu, Y., Byrne, E. M., Zheng, Z., Kemper, K. E., Yengo, L., Mallett, A. J., et al. (2019). Genome-wide association study of medication-use and associated disease in the UK Biobank. *Nat. Commun.* 10, 1891. doi: 10.1038/s41467-019-09572-5.

Yang, W., Li, L., Feng, X., Cheng, H., Ge, X., Bao, Y., et al. (2022). Genome-wide association and Mendelian randomization study of blood copper levels and 213 deep phenotypes in humans. *Commun. Biol.* 5, 405. doi: 10.1038/s42003-022-03351-7.

Yet, I., Menni, C., Shin, S.-Y., Mangino, M., Soranzo, N., Adamski, J., et al. (2016). Genetic Influences on Metabolite Levels: A Comparison across Metabolomic Platforms. *PloS One* 11, e0153672. doi: 10.1371/journal.pone.0153672.
