# Supplementary material for: Non-coding and intergenic genetic variants of human arylamine N-acetyltransferase 2 (NAT2) gene are associated with differential plasma lipid and cholesterol levels and cardiometabolic disorders
Source: Front Pharmacol. 2023 Apr 3;14:1091976. doi: 10.3389/fphar.2023.1091976 (PMC10106703; doi:10.3389/fphar.2023.1091976)
Supplement: Supplementary file 2 [file Table2.docx]

**Supplemental Table 2. *NAT2* genetic variants associated with differential urinary or serum metabolite levels and other traits (Complete list)**

| **Variant** | **Type** | **Risk Allele** | **P-value** | **RAF*** | **Increase/**  **decrease** | **Reported trait** | **Reference** | **Location**† |
| --- | --- | --- | --- | --- | --- | --- | --- | --- |
| rs10109552 | Non-coding | G | 2 x 10^-8^ | 0.69 | NA | Essential tremor | (Müller et al., 2016) | 8:18451300 |
| rs11784251 | Non-coding | G | 2 x 10^-17^ | 0.56 | ↑ | Blood metabolite levels (*N*-acetylputrescine) | (Rhee et al., 2022) | 8:18402503 |
| rs1390360 | Non-coding | A | 7 x 10^-13^ | 0.56 | ↑ | Serum albumin levels | (Sakaue et al., 2021) | 8:18403983 |
| rs146812806 | Non-coding | Ins | 9 x 10^-32^ | 0.35 | ↑ | Serum metabolite levels (5-acetylamino-6-amino-3-methyluracil) | (Feofanova et al., 2020) | 8:18414994 |
|  |  | Ins | 9 x 10^-14^ |  | ↓ | Mean corpuscular hemoglobin concentration | (Chen et al., 2020) |  |
| rs1495741 | Non-coding | NR | 4 x 10^-11^ | - | NA | Bladder cancer | (Rothman et al., 2010) | 8:18415371 |
|  |  | A | 1 x 10^-27^ | 0.65 | ↑ | Blood metabolite levels (1-methylurate) | (Shin et al., 2014) |  |
|  |  | A | 2 x 10^-10^ | . | NA | Bladder cancer | (Figueroa et al., 2014) |  |
|  |  | A | 7 x 10^-11^ |  | NA | Liver injury in anti-tuberculosis drug treatment | (Suvichapanich et al., 2019) |  |
|  |  | A | 1 x 10^-24^ |  | ↓ | *N*-acetylputrescine levels (blood) | (Rhee et al., 2022) |  |
|  |  | A | 2 x 10^-15^ |  | ↓ | 4-acetamidobutanoate levels (blood) | (Rhee et al., 2022) |  |
|  |  | G | 4 x 10^-8^ | 0.35 | NA | Youthful appearance (self-reported) | (Roberts et al., 2020) |  |
|  |  | G | 6 x 10^-21^ |  | ↓ | Urinary metabolite levels in chronic kidney disease | (Schlosser et al., 2020) |  |
| rs1495743 | Non-coding | NR | 6 x 10^-16^ | - | ↓ | Serum metabolite levels (1-methylxanthine) | (Krumsiek et al., 2012) | 8:18415371 |
|  |  | NR | 7 x 10^-9^ | - | ↑ | 4-acetamidobutanoate levels (serum) | (Yet et al., 2016) |  |
|  |  | G | 2 x 10^-40^ | 0.35 | ↓ | Metabolic traits (SM-7 + 11 other traits) | (Suhre et al., 2011a) |  |
|  |  | G | 9 x 10^-14^ |  | ↓ | Iron status biomarkers (total iron binding capacity) | (Bell et al., 2021) |  |
| rs1495745 | Non-coding | T | 9 x 10^-12^ | 0.38 | ↑ | C-reactive protein levels | (Han et al., 2020) | 8:18405213 |
| rs1495747 | Non-coding | C | 3 x 10^-8^ | 0.56 | ↑ | Calcium levels | (Sakaue et al., 2021) | 8:18405351 |
| rs1995003 | Non-coding | T | 6 x 10^-6^ | 0.23 | ↑ | Serum tin levels | (Yang et al., 2022) | 8:18475599 |
| rs35246381 | Non-coding | C | 3 x 10^-35^ | 0.35 | ↑ | Urinary metabolite levels in chronic kidney disease (*N*-acetylputrescine) | (Schlosser et al., 2020) | 8:18415025 |
|  |  | C | 2 x 10^-25^ |  | ↑ | Urinary metabolite modules in chronic kidney disease (4-acetamidobutanoate, allo-threonine, *N*-acetylputrescine) | (Schlosser et al., 2020) |  |
|  |  | C | 1 x 10^-72^ |  | ↑ | Urinary metabolite levels in chronic kidney disease (5-acetylamino-6-formylamino-3-methyluracil) | (Schlosser et al., 2020) |  |
|  |  | C | 7 x 10^-128^ |  | ↑ | Serum metabolite levels (5-acetylamino-6-formylamino-3-methyluracil) | (Feofanova et al., 2020) |  |
|  |  | C | 6 x 10^-24^ |  | ↑ | Urinary metabolite levels in chronic kidney disease (5-acetylamino-6-amino-3-methyluracil) | (Schlosser et al., 2020) |  |
|  |  | C | 3 x 10^-202^ |  | ↑ | Urinary metabolites | (Raffler et al., 2015) |  |
| rs35570672 | Non-coding | T | 4 x 10^-40^ | 0.35 | ↓ | Serum metabolite levels (1-methylxanthine) | (Feofanova et al., 2020) | 8:18415125 |
|  |  | T | 1 x 10^-100^ |  | ↑ | Serum metabolite levels (*N*-acetylputrescine) | (Feofanova et al., 2020) |  |
| rs4646248 | Non-coding | C | 5 x 10^-11^ | 0.39 | ↓ | Liver enzyme levels (alkaline phosphatase) | (Pazoki et al., 2021) | 8:18402845 |
|  |  | T | 2 x 10^-10^ | 0.61 | ↑ | Serum albumin levels | (Sinnott-Armstrong et al., 2021) |  |
| rs4921913 | Non-coding | NR | 7 x 10^-9^ | - | ↓ | 1-methylxanthine levels (serum) | (Yet et al., 2016) | 8:18414867 |
|  |  | NR | 2 x 10^-19^ | - | ↑ | 5-acetylamino-6-formylamino-3-methyluracil levels (serum) | (Bar et al., 2020) |  |
|  |  | C | 6 x 10^-44^ | 0.35 | ↑ | Serum metabolite levels (4-acetamidobutanoate) | (Feofanova et al., 2020) |  |
|  |  | C | 5 x 10^-19^ |  | ↑ | Liver enzyme levels (gamma-glutamyl transferase) | (Pazoki et al., 2021) |  |
| rs1495743 | Non-coding | T | 3 x 10^-47^ | 0.35 | ↓ | Blood metabolite ratios (4-acetamidobutanoate/N1-methyladenosine) | (Shin et al., 2014) | 8:18414867 |
| rs4921914 | Non-coding | NR | 6 x 10^-18^ | - | ↑ | *N*-acetylputrescine levels (serum) | (Bar et al., 2020) | 8:18414928 |
|  |  | C | 1 x 10^-28^ | 0.35 | NA | Urinary metabolites (Formate/succinate ratio) | (Suhre et al., 2011b) |  |
|  |  | C | 5 x 10^-18^ |  | NA | Urinary metabolite levels in chronic kidney disease (X - 12410) | (Schlosser et al., 2020) |  |
|  |  | C | 1 x 10^-11^ |  | ↓ | Urinary metabolite levels in chronic kidney disease (1-methylurate) | (Schlosser et al., 2020) |  |
|  |  | T | 4 x 10^-32^ | 0.65 | ↑ | Urinary metabolites (H-NMR features; unknown) | (Rueedi et al., 2014) |  |
|  |  | T | 1 x 10^-60^ |  | ↑ | Blood metabolite levels (1-methylxanthine) | (Shin et al., 2014) |  |
| rs4921915 | Non-coding | G | 1 x 10^-19^ | 0.35 | ↑ | Urinary metabolite levels in chronic kidney disease (4-acetamidobutanoate) | (Schlosser et al., 2020) | 8:18414956 |
| rs66477371 | Non-coding | T | 6 x 10^-6^ | 0.07 | ↓ | Academic attainment (math) | (Donati et al., 2021) | 8:18456207 |
| rs7006687 | Non-coding | T | 2 x 10^-6^ | 0.57 | NA | QT interval (drug interaction; sulfonylurea hypoglycemic agents) | (Avery et al., 2014) | 8:18376073 |
| rs721399 | Non-coding | T | 4 x 10^-58^ | 0.56 | ↓ | Blood metabolite levels (4-acetamidobutanoate) | (Shin et al., 2014) | 8:18401856 |
|  |  | T | 2 x 10^-10^ |  | ↓ | 4-acetamidobutanoate levels (blood) | (Rhee et al., 2022) |  |
|  |  |  |  |  |  |  |  |  |

*, Based on 1000 Genomes Project (phase 3)

†, Human GRCh38/hg38

RAF, relative allele frequency. NR, not reported. NA, not available.

Ins, insertion

**References**

Avery, C. L., Sitlani, C. M., Arking, D. E., Arnett, D. K., Bis, J. C., Boerwinkle, E., et al. (2014). Drug-gene interactions and the search for missing heritability: a cross-sectional pharmacogenomics study of the QT interval. *Pharmacogenomics J.* 14, 6–13. doi: 10.1038/tpj.2013.4.

Bar, N., Korem, T., Weissbrod, O., Zeevi, D., Rothschild, D., Leviatan, S., et al. (2020). A reference map of potential determinants for the human serum metabolome. *Nature* 588, 135–140. doi: 10.1038/s41586-020-2896-2.

Bell, S., Rigas, A. S., Magnusson, M. K., Ferkingstad, E., Allara, E., Bjornsdottir, G., et al. (2021). A genome-wide meta-analysis yields 46 new loci associating with biomarkers of iron homeostasis. *Commun. Biol.* 4, 156. doi: 10.1038/s42003-020-01575-z.

Chen, M.-H., Raffield, L. M., Mousas, A., Sakaue, S., Huffman, J. E., Moscati, A., et al. (2020). Trans-ethnic and Ancestry-Specific Blood-Cell Genetics in 746,667 Individuals from 5 Global Populations. *Cell* 182, 1198-1213.e14. doi: 10.1016/j.cell.2020.06.045.

Donati, G., Dumontheil, I., Pain, O., Asbury, K., and Meaburn, E. L. (2021). Evidence for specificity of polygenic contributions to attainment in English, maths and science during adolescence. *Sci. Rep.* 11, 3851. doi: 10.1038/s41598-021-82877-y.

Feofanova, E. V., Chen, H., Dai, Y., Jia, P., Grove, M. L., Morrison, A. C., et al. (2020). A Genome-wide Association Study Discovers 46 Loci of the Human Metabolome in the Hispanic Community Health Study/Study of Latinos. *Am. J. Hum. Genet.* 107, 849–863. doi: 10.1016/j.ajhg.2020.09.003.

Figueroa, J. D., Ye, Y., Siddiq, A., Garcia-Closas, M., Chatterjee, N., Prokunina-Olsson, L., et al. (2014). Genome-wide association study identifies multiple loci associated with bladder cancer risk. *Hum. Mol. Genet.* 23, 1387–1398. doi: 10.1093/hmg/ddt519.

Han, X., Ong, J.-S., An, J., Hewitt, A. W., Gharahkhani, P., and MacGregor, S. (2020). Using Mendelian randomization to evaluate the causal relationship between serum C-reactive protein levels and age-related macular degeneration. *Eur. J. Epidemiol.* 35, 139–146. doi: 10.1007/s10654-019-00598-z.

Krumsiek, J., Suhre, K., Evans, A. M., Mitchell, M. W., Mohney, R. P., Milburn, M. V., et al. (2012). Mining the unknown: a systems approach to metabolite identification combining genetic and metabolic information. *PLoS Genet.* 8, e1003005. doi: 10.1371/journal.pgen.1003005.

Müller, S. H., Girard, S. L., Hopfner, F., Merner, N. D., Bourassa, C. V., Lorenz, D., et al. (2016). Genome-wide association study in essential tremor identifies three new loci. *Brain J. Neurol.* 139, 3163–3169. doi: 10.1093/brain/aww242.

Pazoki, R., Vujkovic, M., Elliott, J., Evangelou, E., Gill, D., Ghanbari, M., et al. (2021). Genetic analysis in European ancestry individuals identifies 517 loci associated with liver enzymes. *Nat. Commun.* 12, 2579. doi: 10.1038/s41467-021-22338-2.

Raffler, J., Friedrich, N., Arnold, M., Kacprowski, T., Rueedi, R., Altmaier, E., et al. (2015). Genome-Wide Association Study with Targeted and Non-targeted NMR Metabolomics Identifies 15 Novel Loci of Urinary Human Metabolic Individuality. *PLoS Genet.* 11, e1005487. doi: 10.1371/journal.pgen.1005487.

Rhee, E. P., Surapaneni, A., Zheng, Z., Zhou, L., Dutta, D., Arking, D. E., et al. (2022). Trans-ethnic genome-wide association study of blood metabolites in the Chronic Renal Insufficiency Cohort (CRIC) study. *Kidney Int.* 101, 814–823. doi: 10.1016/j.kint.2022.01.014.

Roberts, V., Main, B., Timpson, N. J., and Haworth, S. (2020). Genome-Wide Association Study Identifies Genetic Associations with Perceived Age. *J. Invest. Dermatol.* 140, 2380–2385. doi: 10.1016/j.jid.2020.03.970.

Rothman, N., Garcia-Closas, M., Chatterjee, N., Malats, N., Wu, X., Figueroa, J. D., et al. (2010). A multi-stage genome-wide association study of bladder cancer identifies multiple susceptibility loci. *Nat. Genet.* 42, 978–984. doi: 10.1038/ng.687.

Rueedi, R., Ledda, M., Nicholls, A. W., Salek, R. M., Marques-Vidal, P., Morya, E., et al. (2014). Genome-wide association study of metabolic traits reveals novel gene-metabolite-disease links. *PLoS Genet.* 10, e1004132. doi: 10.1371/journal.pgen.1004132.

Sakaue, S., Kanai, M., Tanigawa, Y., Karjalainen, J., Kurki, M., Koshiba, S., et al. (2021). A cross-population atlas of genetic associations for 220 human phenotypes. *Nat. Genet.* 53, 1415–1424. doi: 10.1038/s41588-021-00931-x.

Schlosser, P., Li, Y., Sekula, P., Raffler, J., Grundner-Culemann, F., Pietzner, M., et al. (2020). Genetic studies of urinary metabolites illuminate mechanisms of detoxification and excretion in humans. *Nat. Genet.* 52, 167–176. doi: 10.1038/s41588-019-0567-8.

Shin, S.-Y., Fauman, E. B., Petersen, A.-K., Krumsiek, J., Santos, R., Huang, J., et al. (2014). An atlas of genetic influences on human blood metabolites. *Nat. Genet.* 46, 543–550. doi: 10.1038/ng.2982.

Sinnott-Armstrong, N., Tanigawa, Y., Amar, D., Mars, N., Benner, C., Aguirre, M., et al. (2021). Genetics of 35 blood and urine biomarkers in the UK Biobank. *Nat. Genet.* 53, 185–194. doi: 10.1038/s41588-020-00757-z.

Suhre, K., Shin, S.-Y., Petersen, A.-K., Mohney, R. P., Meredith, D., Wägele, B., et al. (2011a). Human metabolic individuality in biomedical and pharmaceutical research. *Nature* 477, 54–60. doi: 10.1038/nature10354.

Suhre, K., Wallaschofski, H., Raffler, J., Friedrich, N., Haring, R., Michael, K., et al. (2011b). A genome-wide association study of metabolic traits in human urine. *Nat. Genet.* 43, 565–569. doi: 10.1038/ng.837.

Suvichapanich, S., Wattanapokayakit, S., Mushiroda, T., Yanai, H., Chuchottawon, C., Kantima, T., et al. (2019). Genomewide Association Study Confirming the Association of NAT2 with Susceptibility to Antituberculosis Drug-Induced Liver Injury in Thai Patients. *Antimicrob. Agents Chemother.* 63, e02692-18. doi: 10.1128/AAC.02692-18.

Wu, Y., Byrne, E. M., Zheng, Z., Kemper, K. E., Yengo, L., Mallett, A. J., et al. (2019). Genome-wide association study of medication-use and associated disease in the UK Biobank. *Nat. Commun.* 10, 1891. doi: 10.1038/s41467-019-09572-5.

Yang, W., Li, L., Feng, X., Cheng, H., Ge, X., Bao, Y., et al. (2022). Genome-wide association and Mendelian randomization study of blood copper levels and 213 deep phenotypes in humans. *Commun. Biol.* 5, 405. doi: 10.1038/s42003-022-03351-7.

Yet, I., Menni, C., Shin, S.-Y., Mangino, M., Soranzo, N., Adamski, J., et al. (2016). Genetic Influences on Metabolite Levels: A Comparison across Metabolomic Platforms. *PloS One* 11, e0153672. doi: 10.1371/journal.pone.0153672.
